# Supplementary material for: An Exploratory Study of the Enzymatic Hydroxycinnamoylation of Sucrose and Its Derivatives
Source: Molecules. 2024 Aug 28;29(17):4067. doi: 10.3390/molecules29174067 (PMC11397724; doi:10.3390/molecules29174067)

Supplementary materials

An Exploratory Study of the Enzymatic Hydroxycinnamoylation of Sucrose and Its Derivatives

Matej Cvečko [1], Vladimír Mastihuba and Mária Mastihubová *

Institute of Chemistry, Slovak Academy of Sciences, 845 38 Bratislava, Slovakia; matej.cvecko@savba.sk (M.C.); vladimir.mastihuba@savba.sk (V.M.)
* Correspondence: maria.mastihubova@savba.sk; Tel.: +421-2-59410655

^{1}H and ^{13}C NMR spectra of sucroses acylated with substituted cinnamates

Figures S1 – S36

Figure S1. ^{1}H NMR of 6,1'-di-*O*-feruloyl sucrose (**6c**)

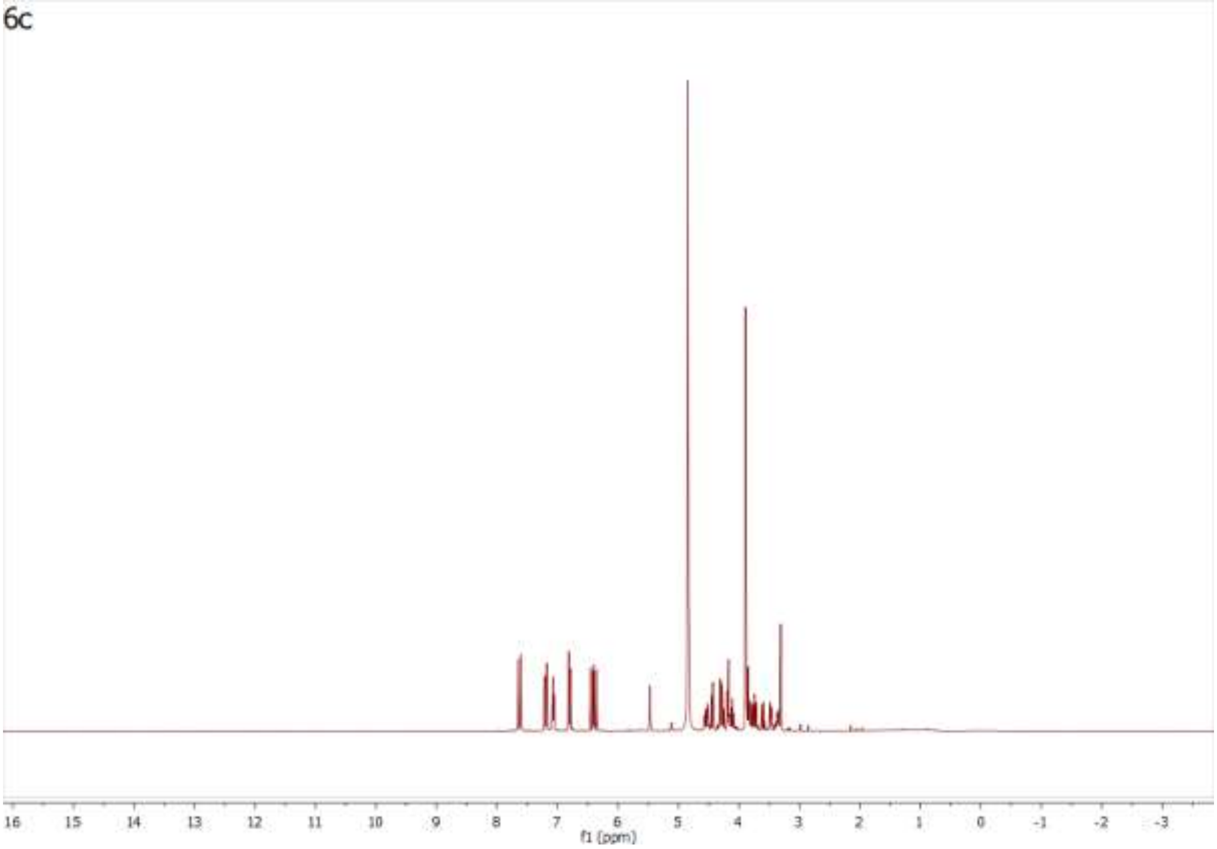

Figure S2. ^{13}C NMR of 6,1'-di-*O*-feruloyl sucrose (**6c**)

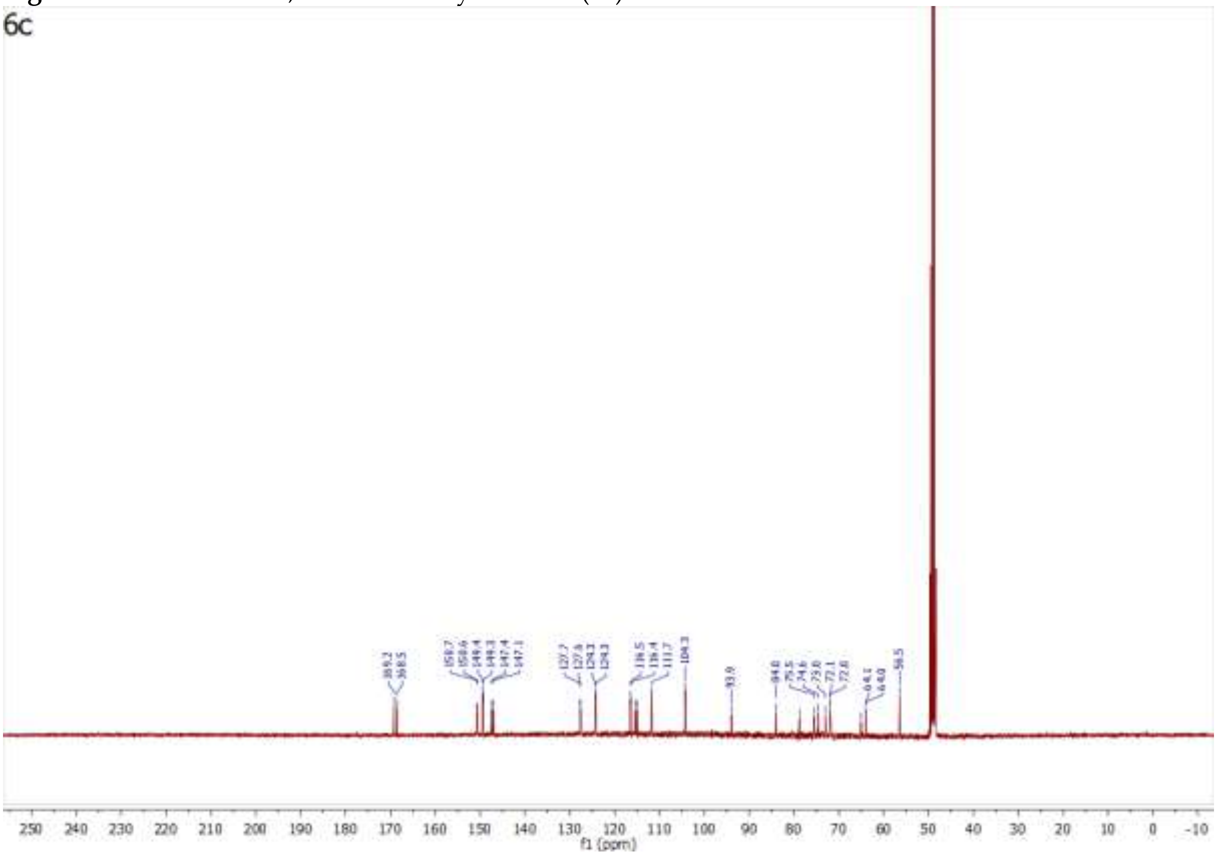

Figure S3. ^{1}H NMR of 6'-*O*-coumaroyl-2,1':4,6-di-*O*-isopropylidene sucrose (**9a**)

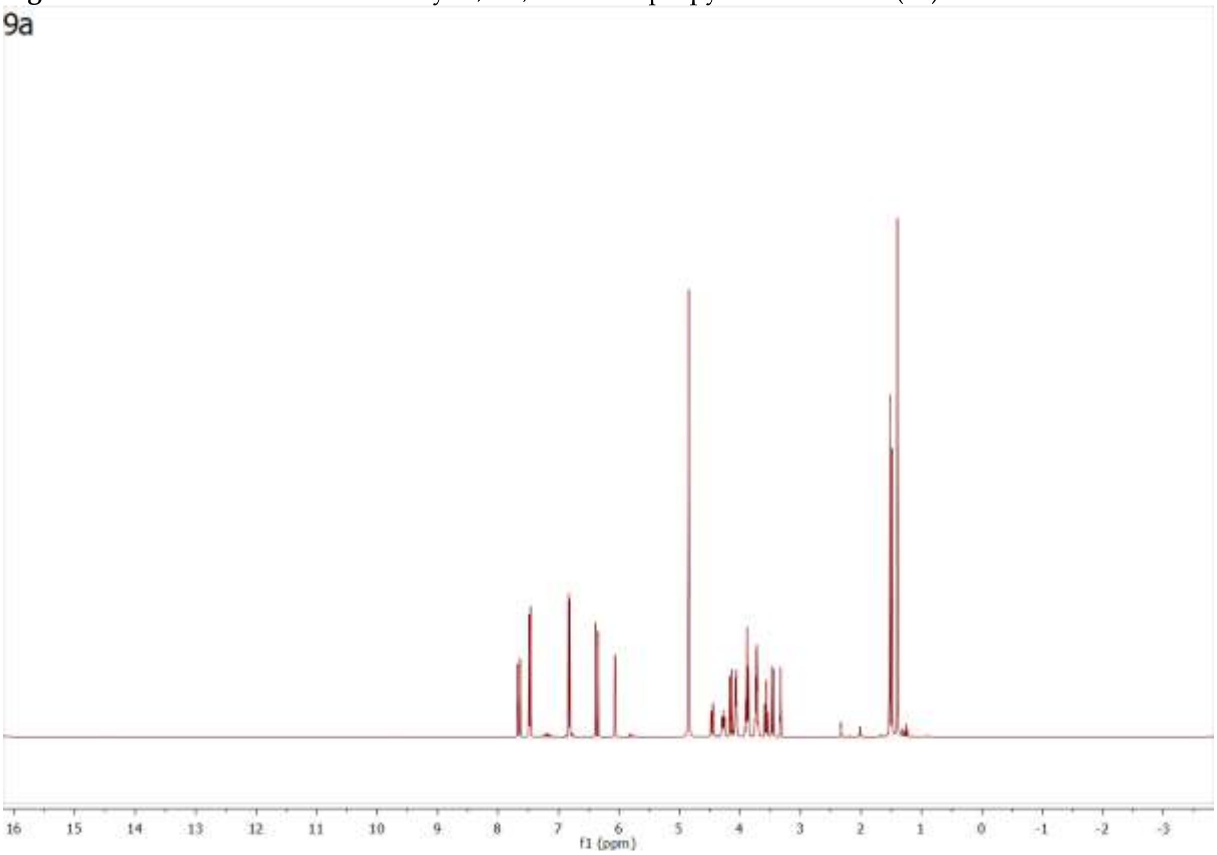

Figure S4. ^{13}C NMR of 6'-*O*-coumaroyl-2,1':4,6-di-*O*-isopropylidene sucrose (**9a**)

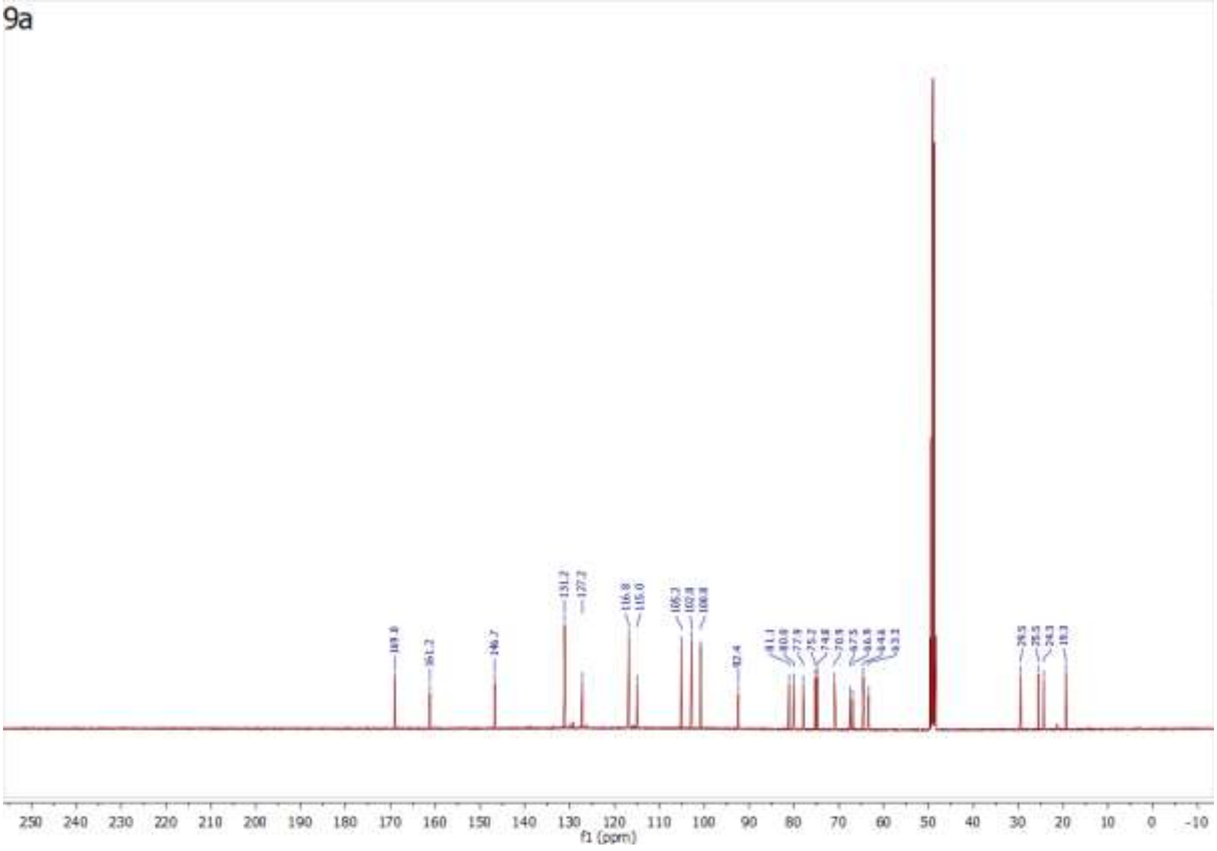

Figure S5. ^{1}H NMR of 6'-*O*-feruloyl-2,1':4,6-di-*O*-isopropylidene sucrose (**9b**)

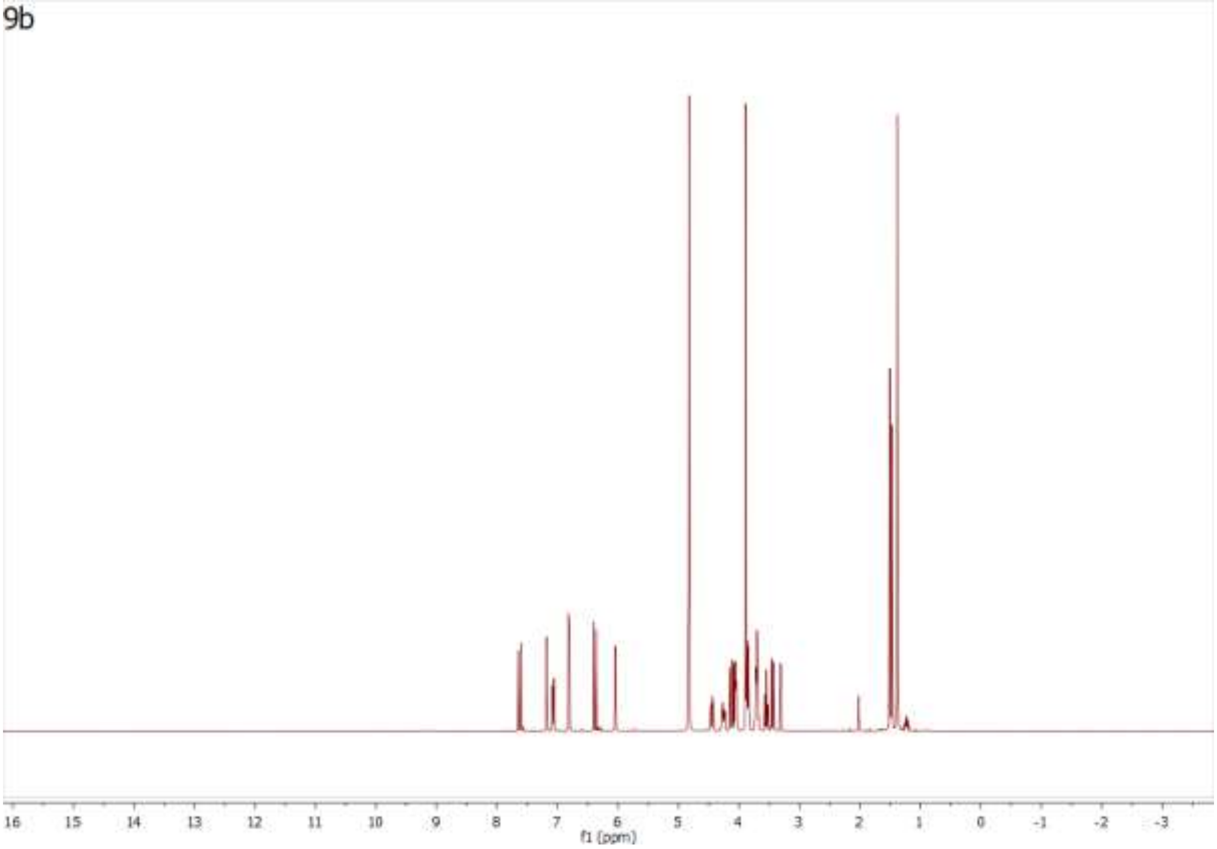

Figure S6. ^{13}C NMR of 6'-*O*-feruloyl-2,1':4,6-di-*O*-isopropylidene sucrose (**9b**)

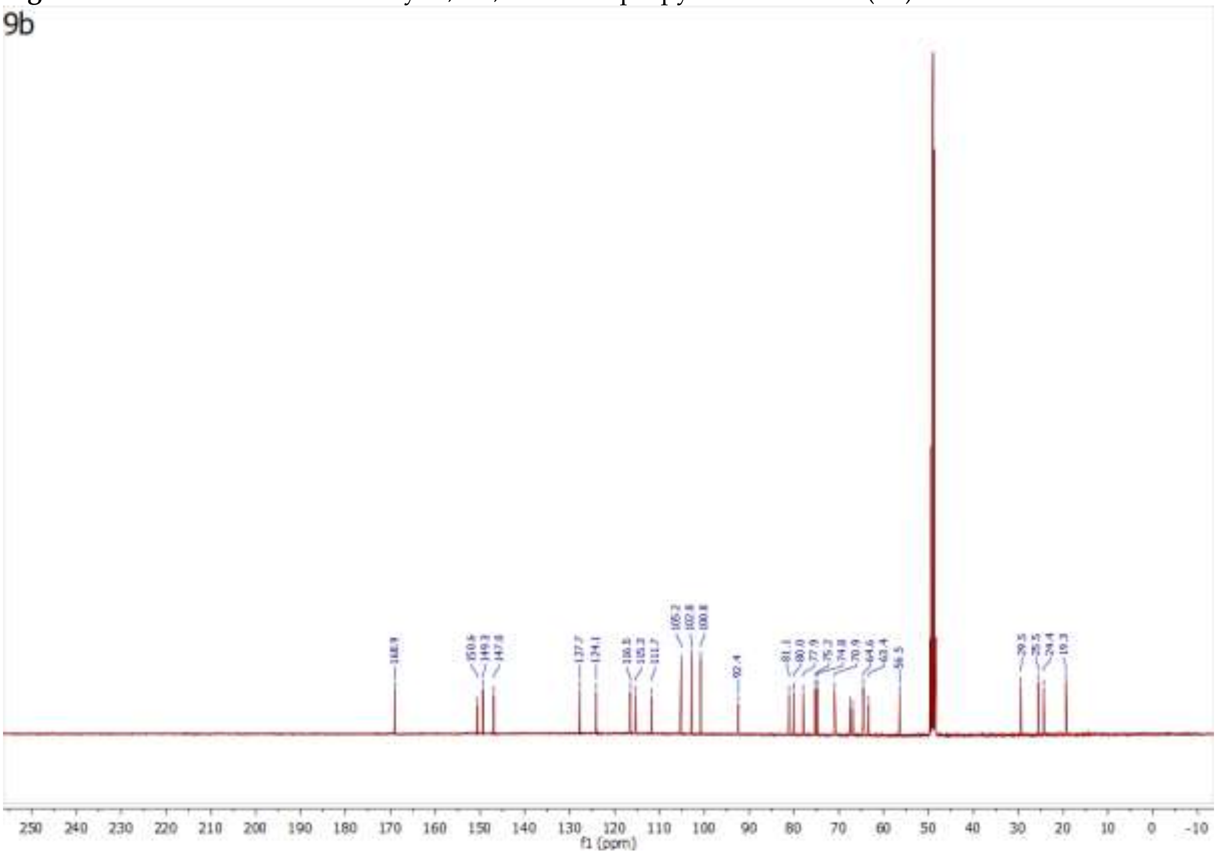

Figure S7. ^{1}H NMR of 1′-*O*-coumaroyl-4,6-*O*-isopropylidene sucrose (**10a**)

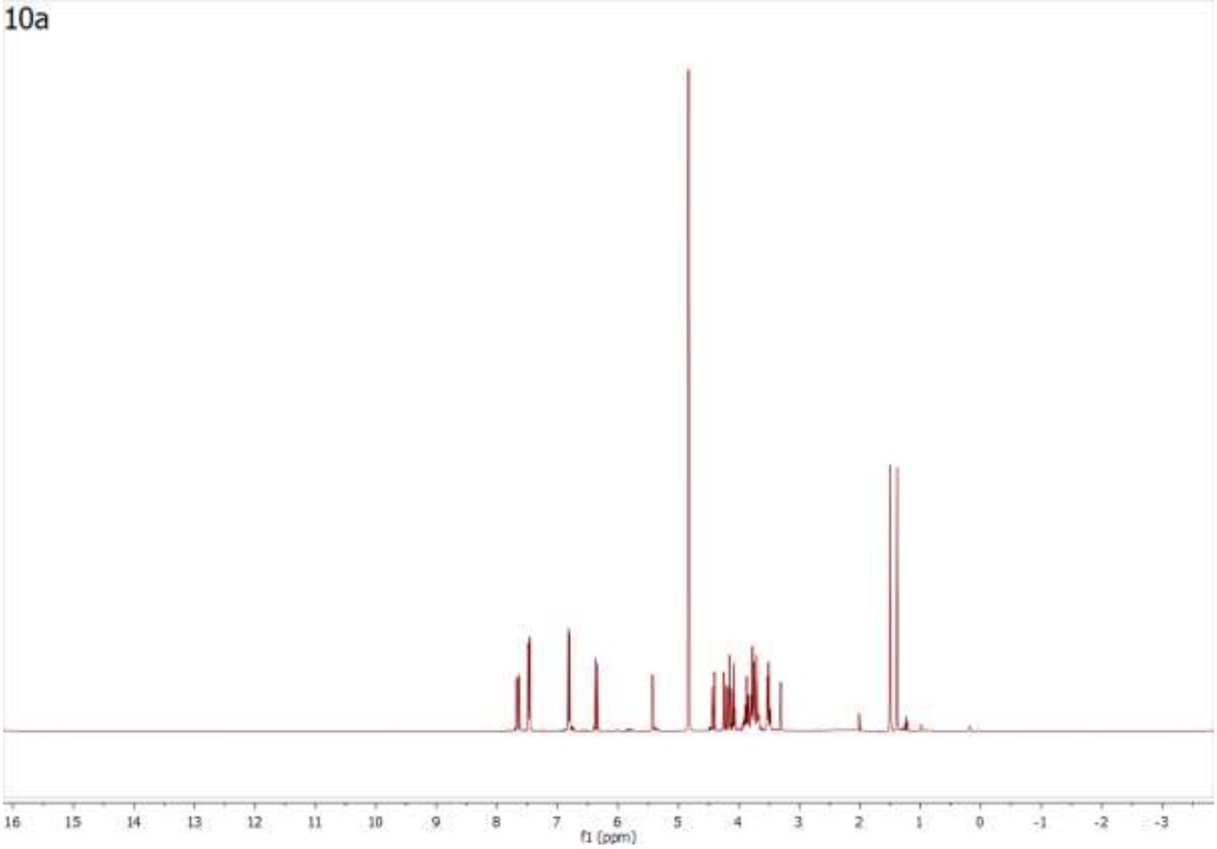

Figure S8. ^{13}C NMR of 1′-*O*-coumaroyl-4,6-*O*-isopropylidene sucrose (**10a**)

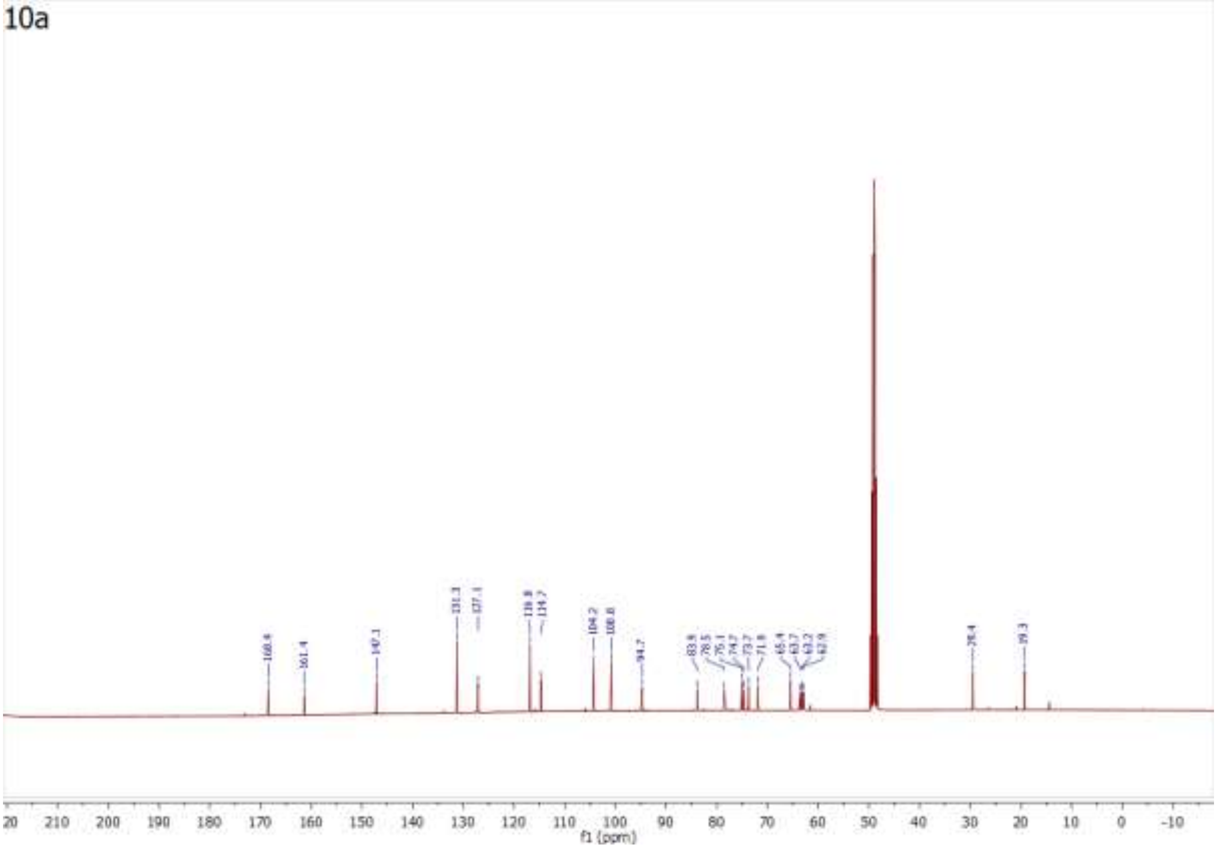

Figure S9. ^{1}H NMR of 1′-*O*-feruloyl-4,6-*O*-isopropylidene sucrose (**10b**)

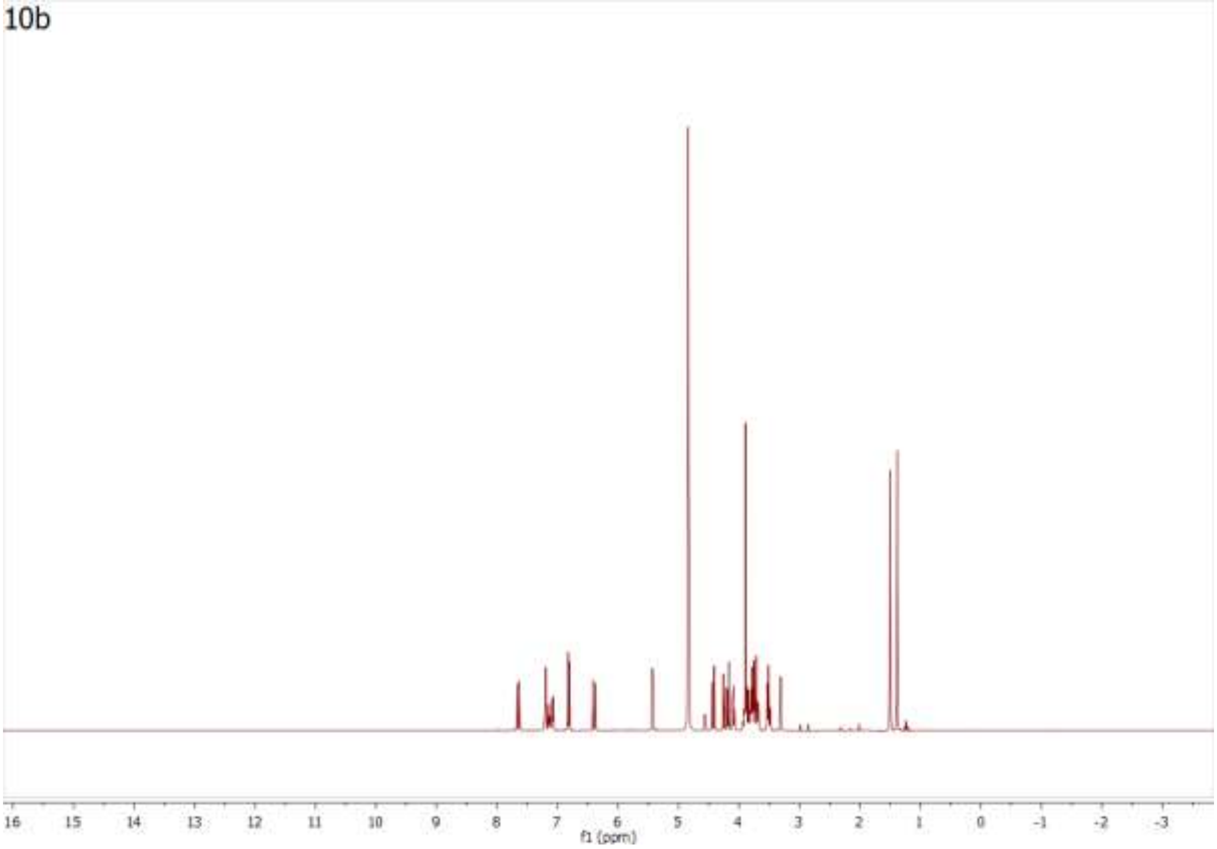

Figure S10. ^{13}C NMR of 1′-*O*-feruloyl-4,6-*O*-isopropylidene sucrose (**10b**)

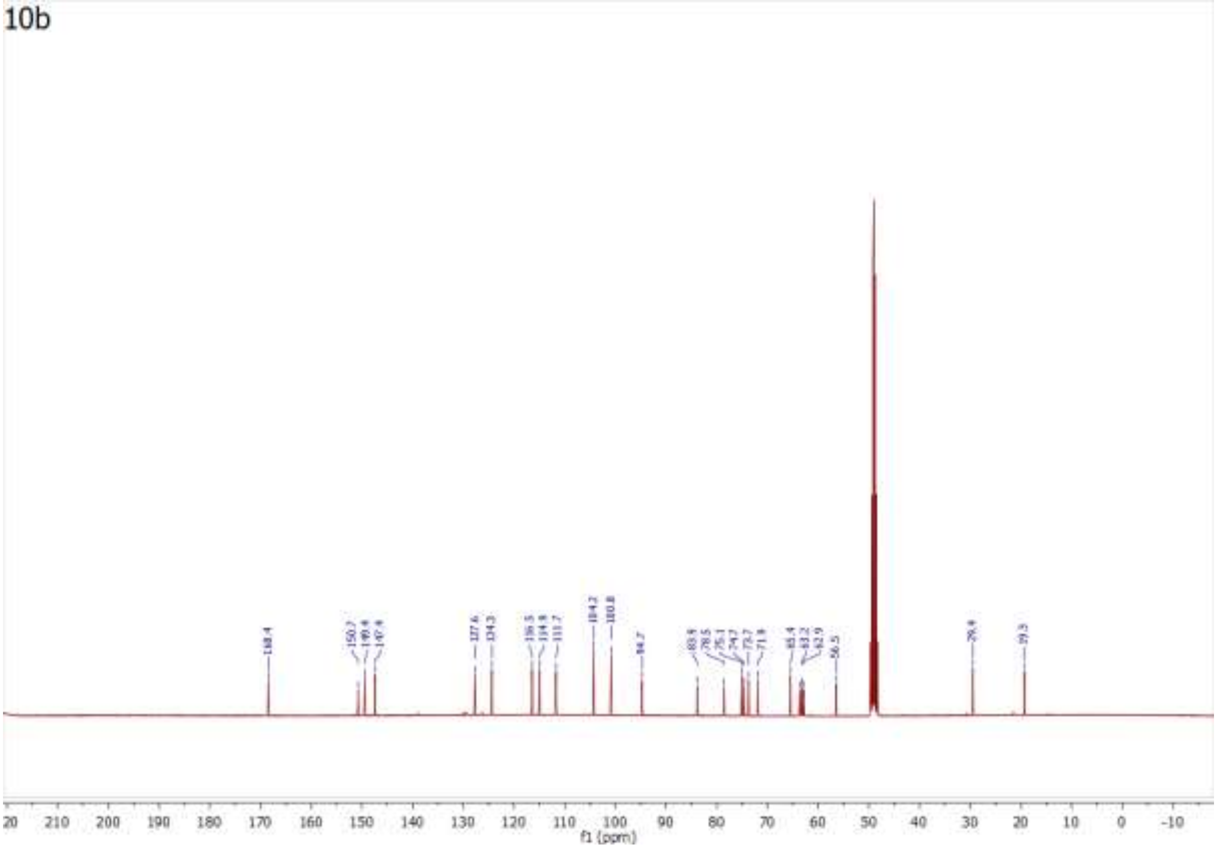

Figure S11. ^{1}H NMR of 4′-*O*-coumaroyl-4,6-*O*-isopropylidene sucrose (**11a**)

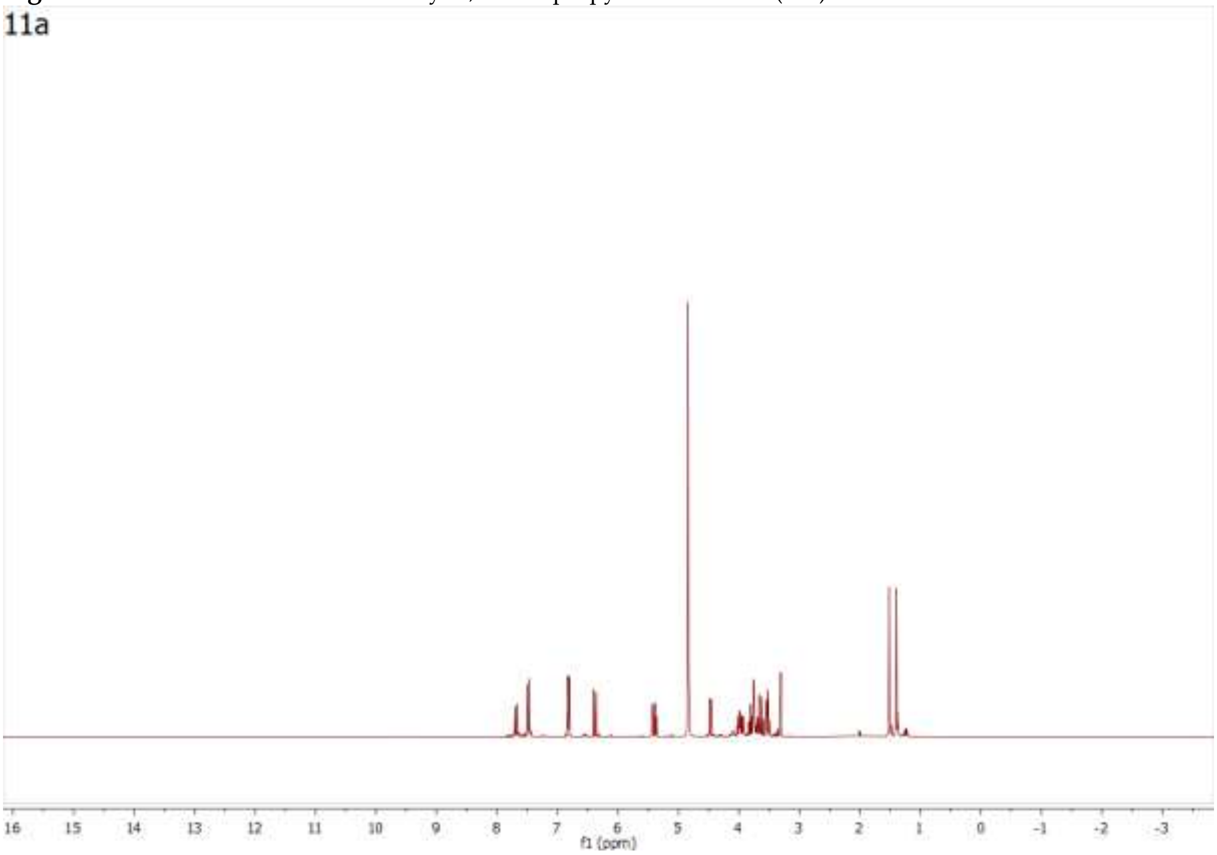

Figure S12. ^{13}C NMR of 4′-*O*-coumaroyl-4,6-*O*-isopropylidene sucrose (**11a**)

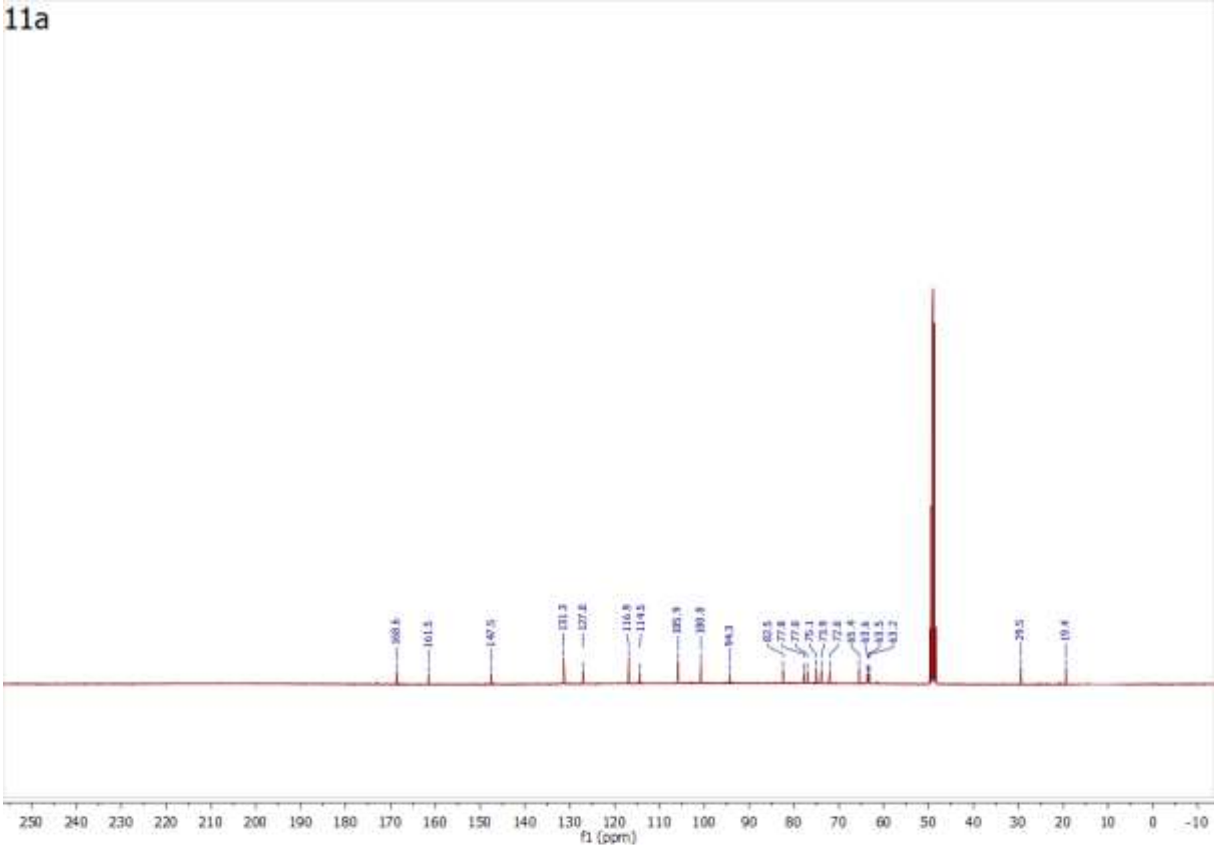

Figure S13. ^{1}H NMR of 6′-*O*-coumaroyl-4,6-*O*-isopropylidene sucrose (**12a**)

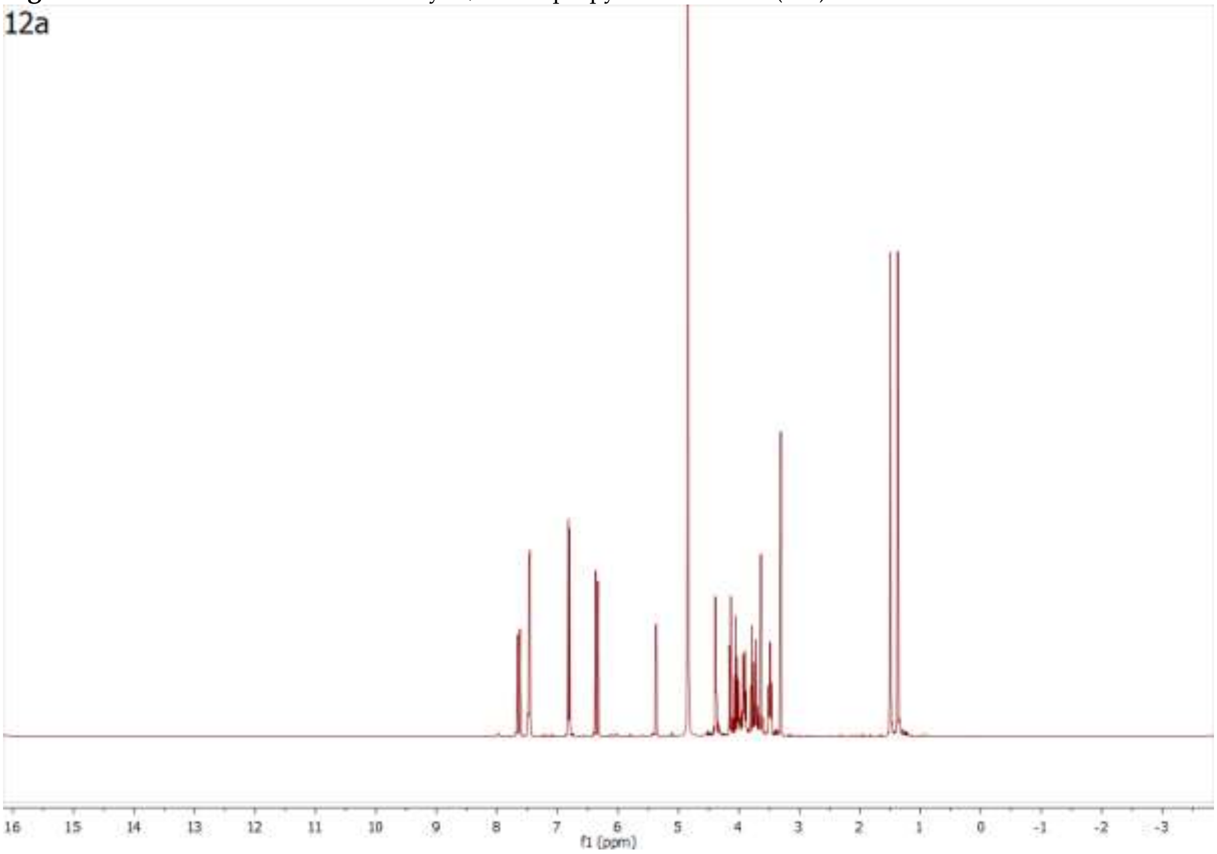

Figure S14. ^{13}C NMR of 6′-*O*-coumaroyl-4,6-*O*-isopropylidene sucrose (**12a**)

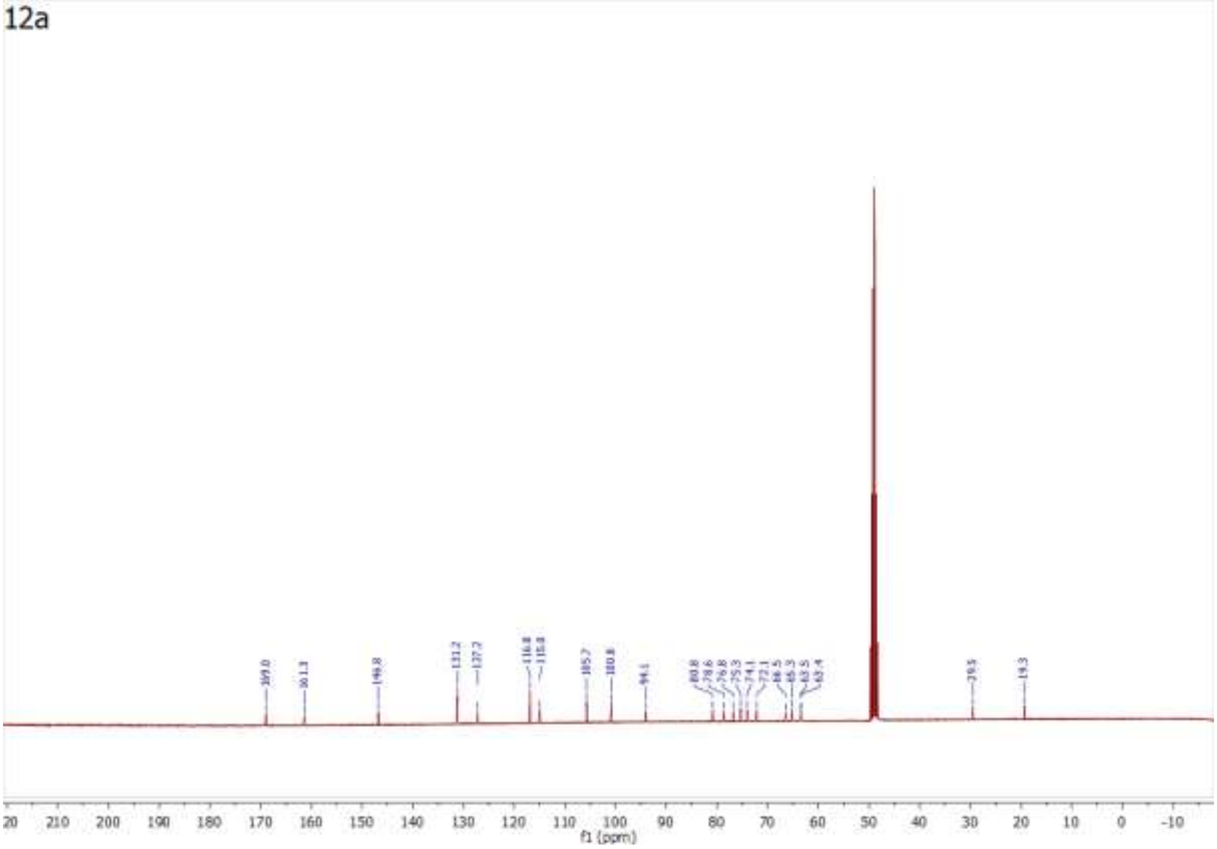

Figure S15. ^{1}H NMR of 6′-*O*-feruloyl-4,6-*O*-isopropylidene sucrose (**12b**)

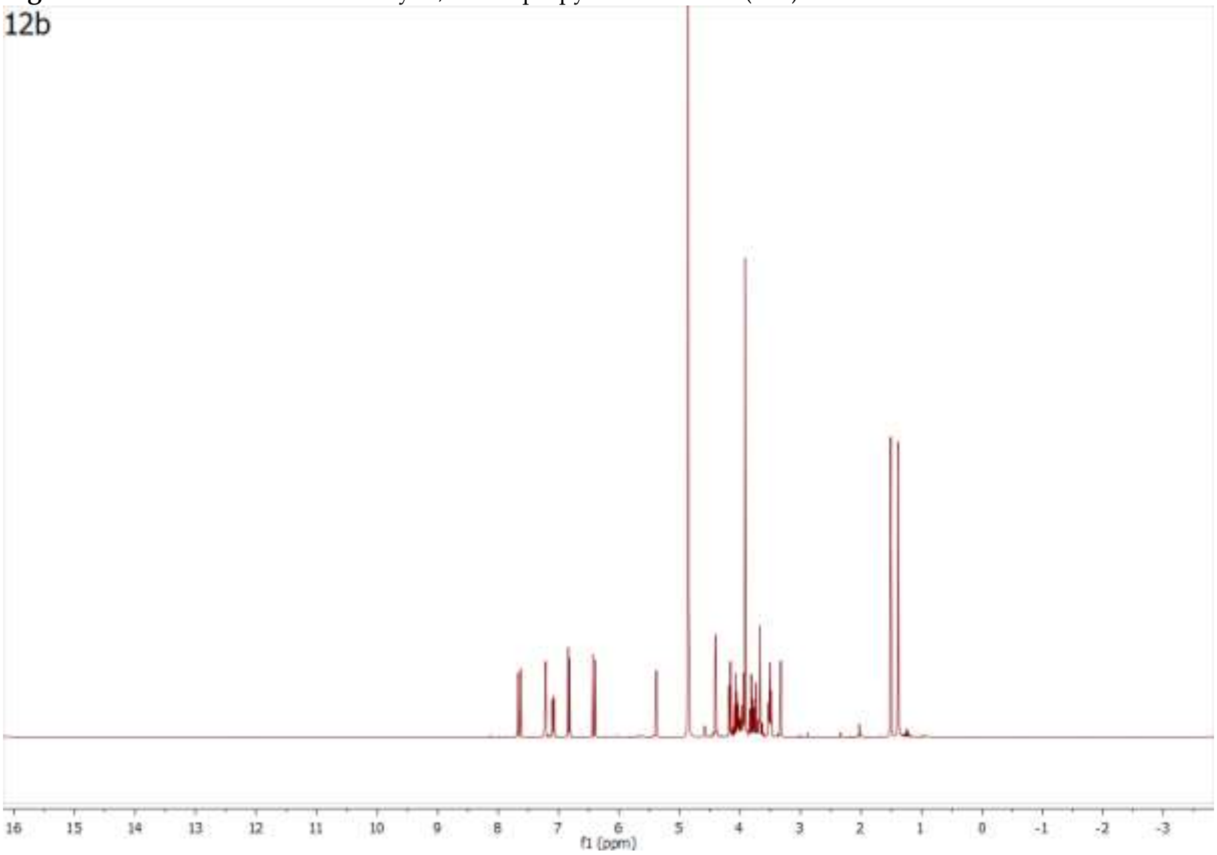

Figure S16. ^{13}C NMR of 6′-*O*-feruloyl-4,6-*O*-isopropylidene sucrose (**12b**)

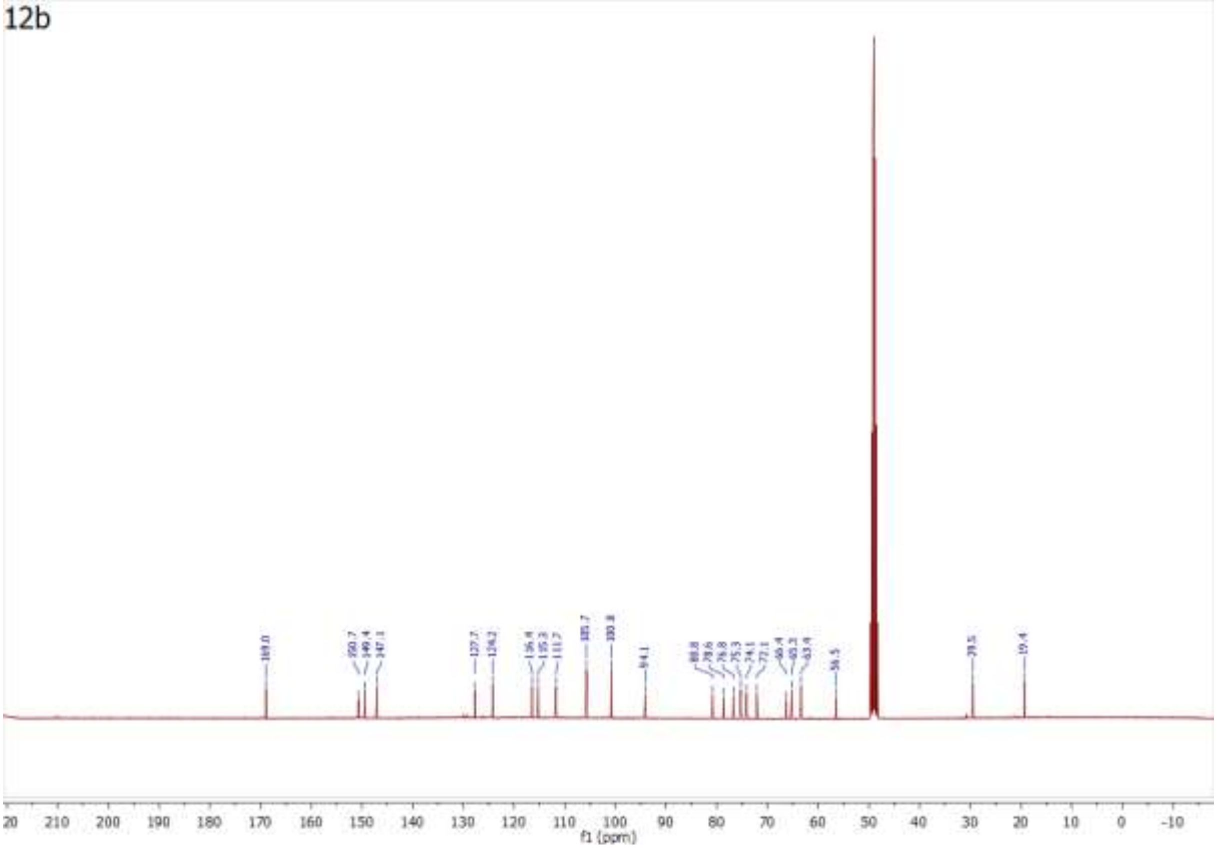

Figure S17. ^{1}H NMR of 1′,4′-di-*O*-coumaroyl-4,6-*O*-isopropylidene sucrose (**13a**)

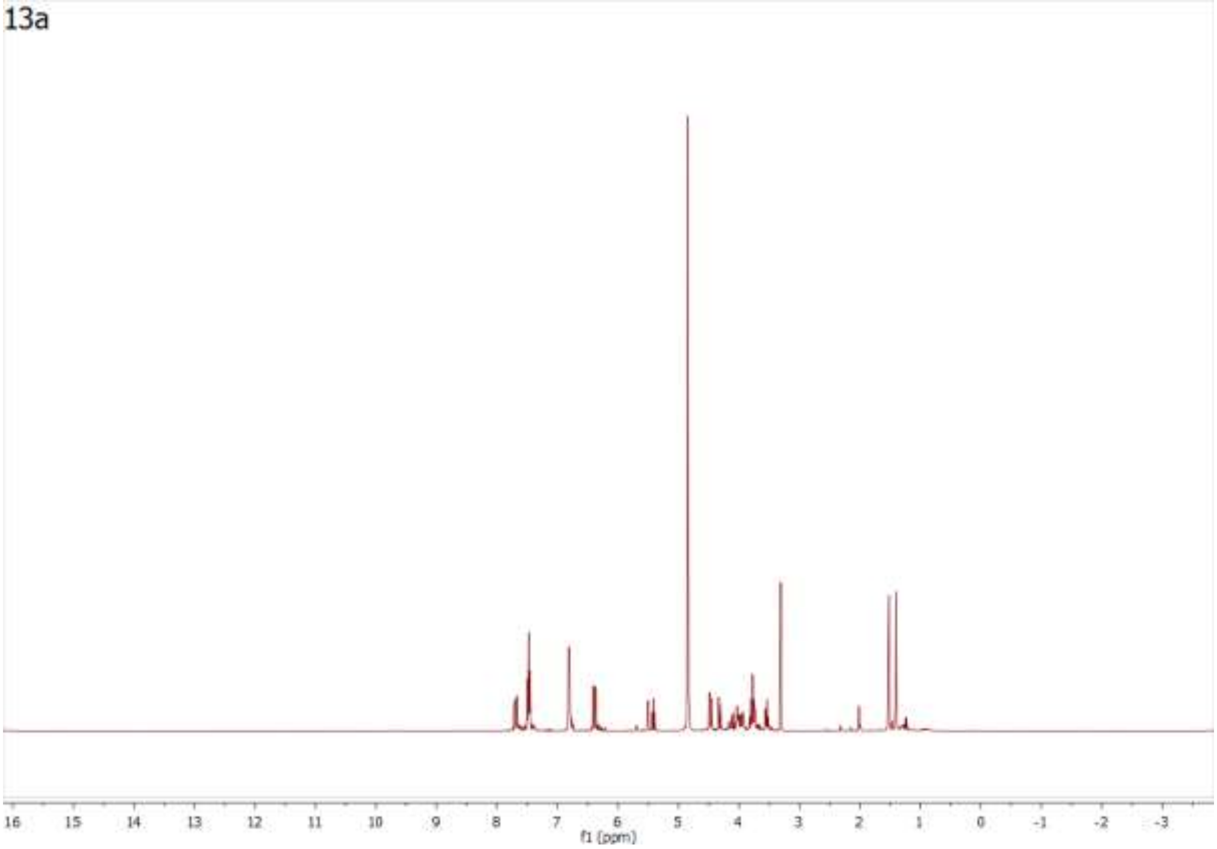

Figure S18. ^{13}C NMR of 1′,4′-di-*O*-coumaroyl-4,6-*O*-isopropylidene sucrose (**13a**)

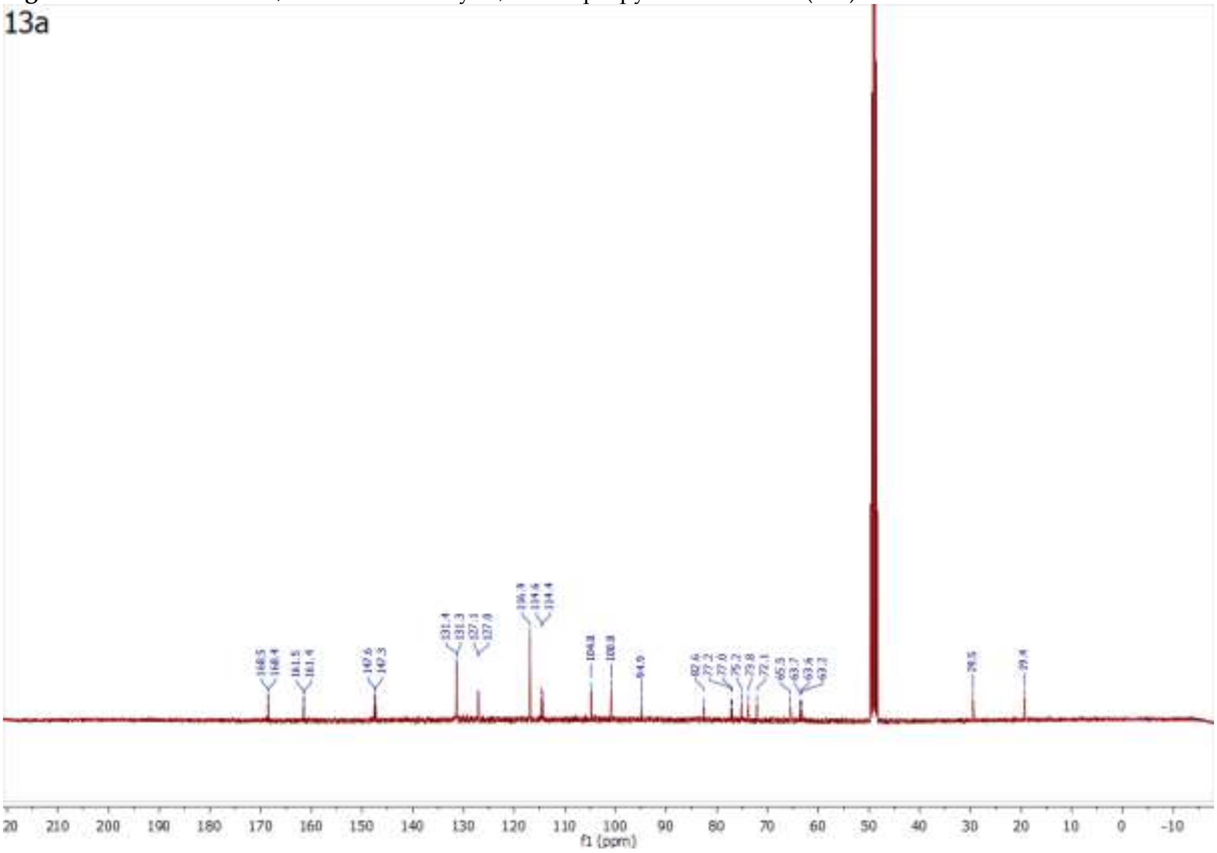

Figure S19. ^{1}H NMR of 1′,6′-di-*O*-coumaroyl-4,6-*O*-isopropylidene sucrose (**14a**)

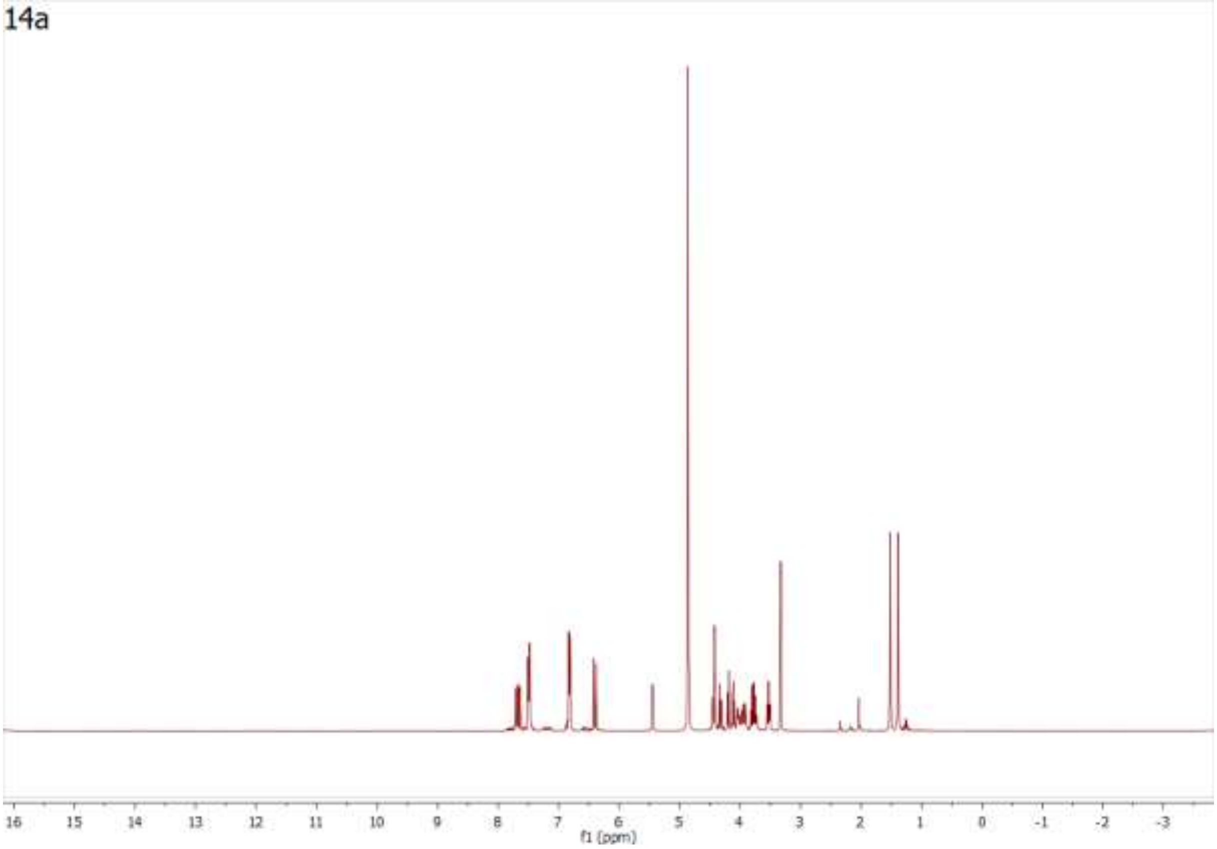

Figure S20. ^{13}C NMR of 1′,6′-di-*O*-coumaroyl-4,6-*O*-isopropylidene sucrose (**14a**)

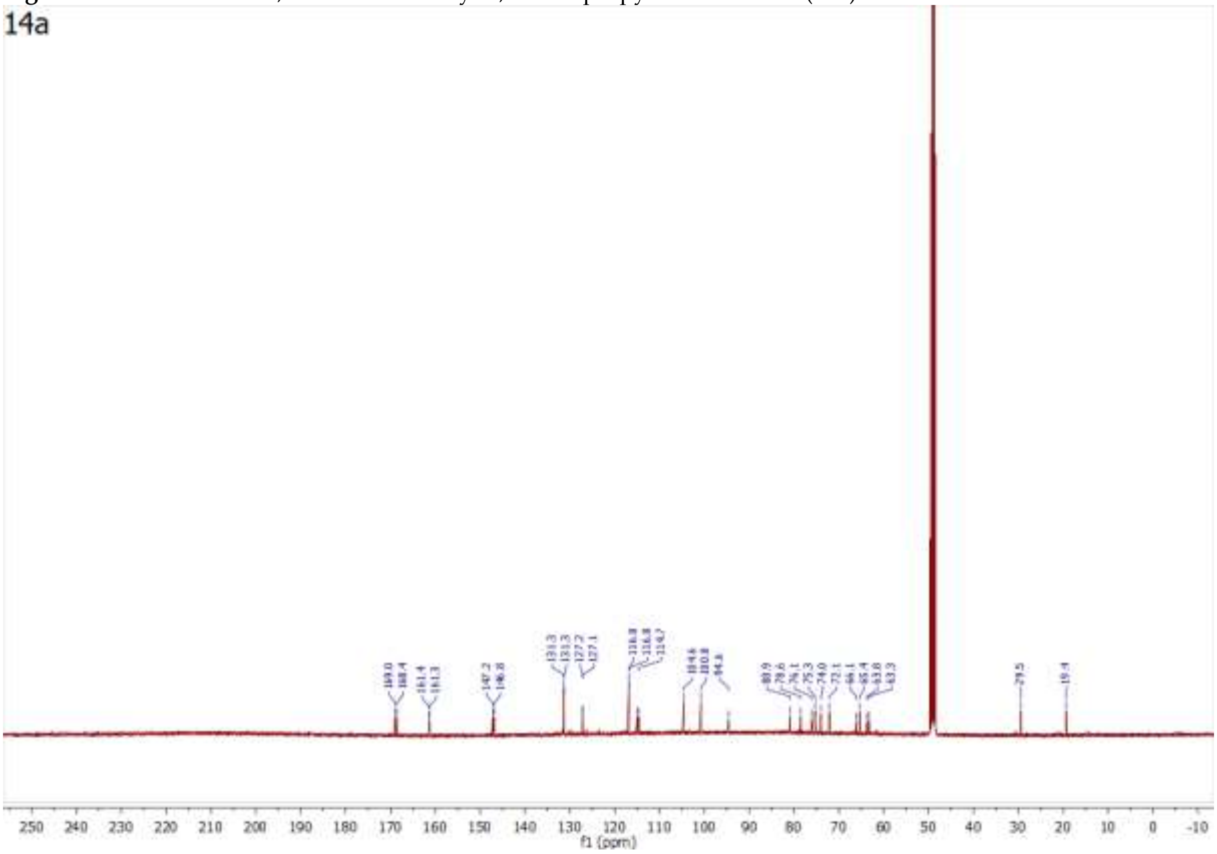

Figure S21. ^{1}H NMR of 1′,6′-di-*O*-feruloyl-4,6-*O*-isopropylidene sucrose (**14b**)

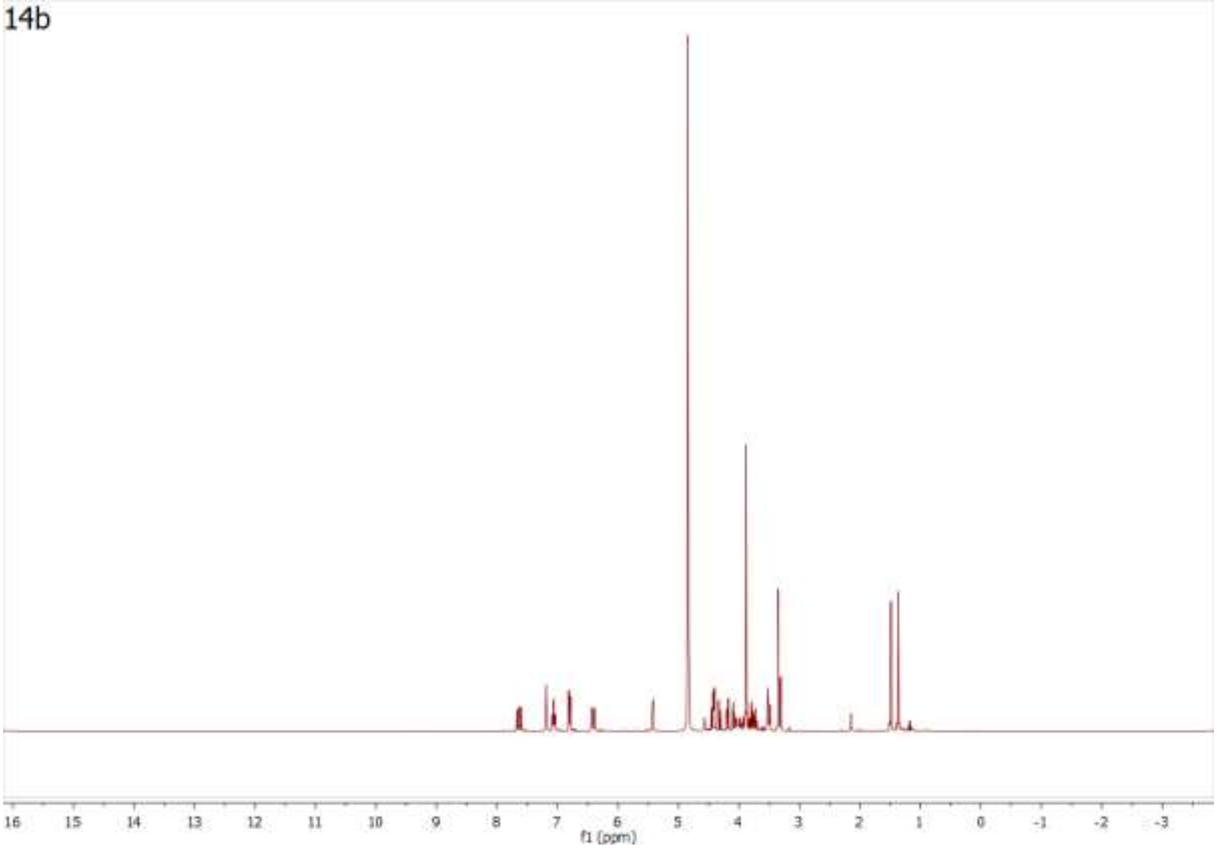

Figure S22. ^{13}C NMR of 1′,6′-di-*O*-feruloyl-4,6-*O*-isopropylidene sucrose (**14b**)

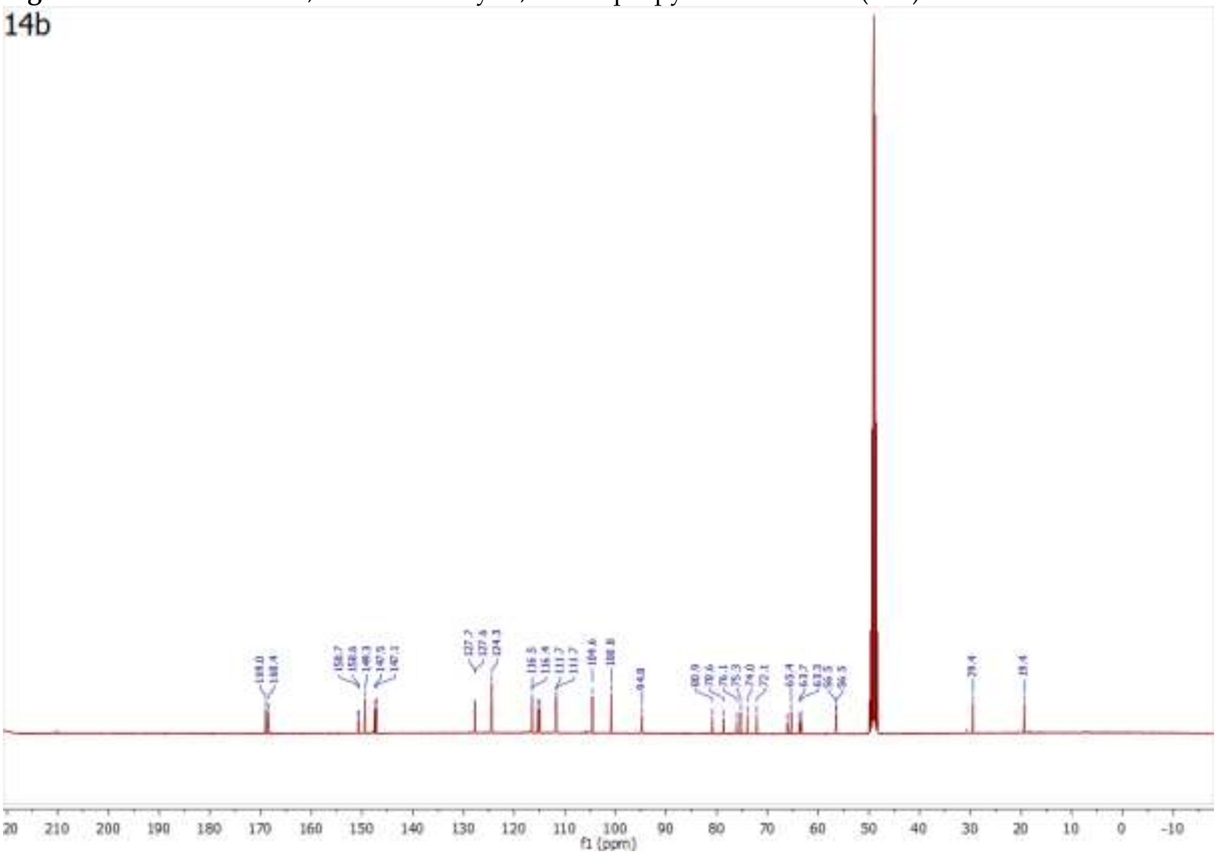

Figure S23. ^{1}H NMR of 6,3'-di-*O*-coumaroyl sucrose (**15a**)

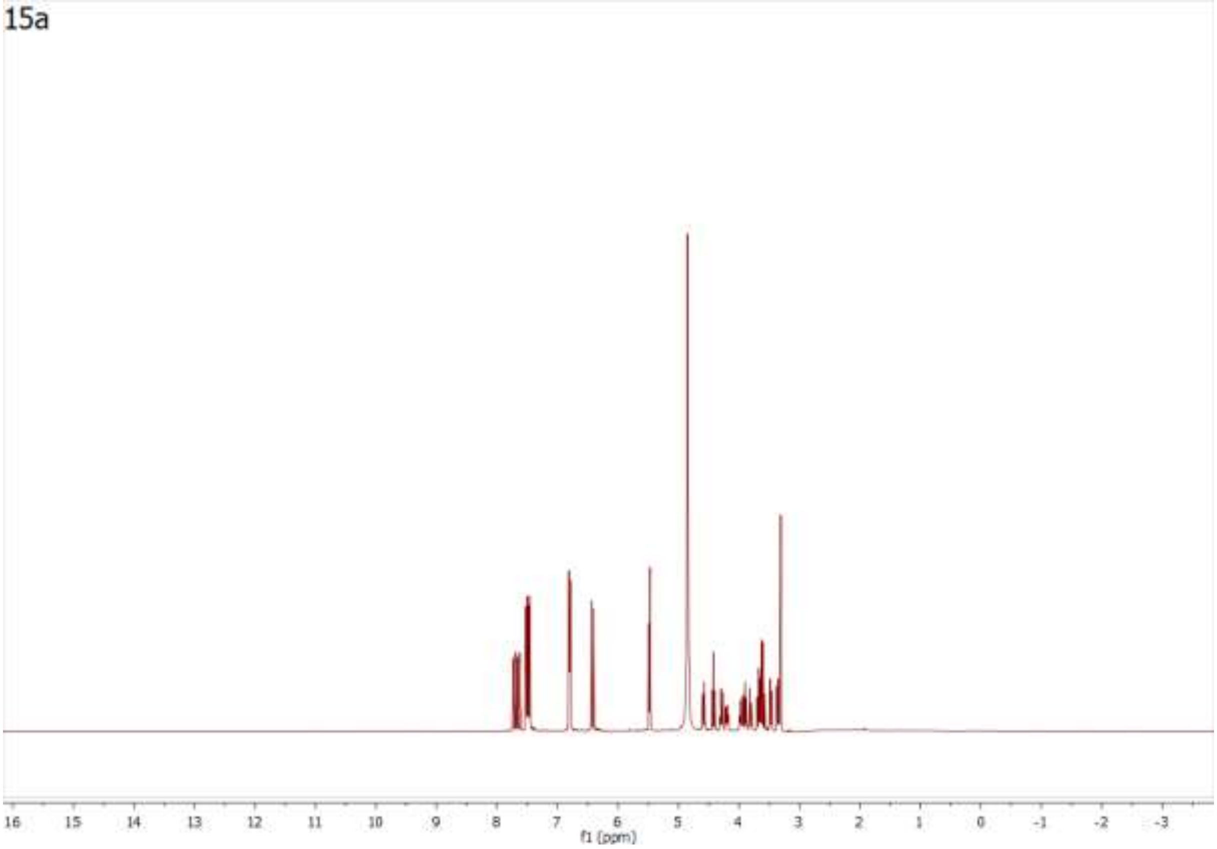

Figure S24. ^{13}C NMR of 6,3'-di-*O*-coumaroyl sucrose (**15a**)

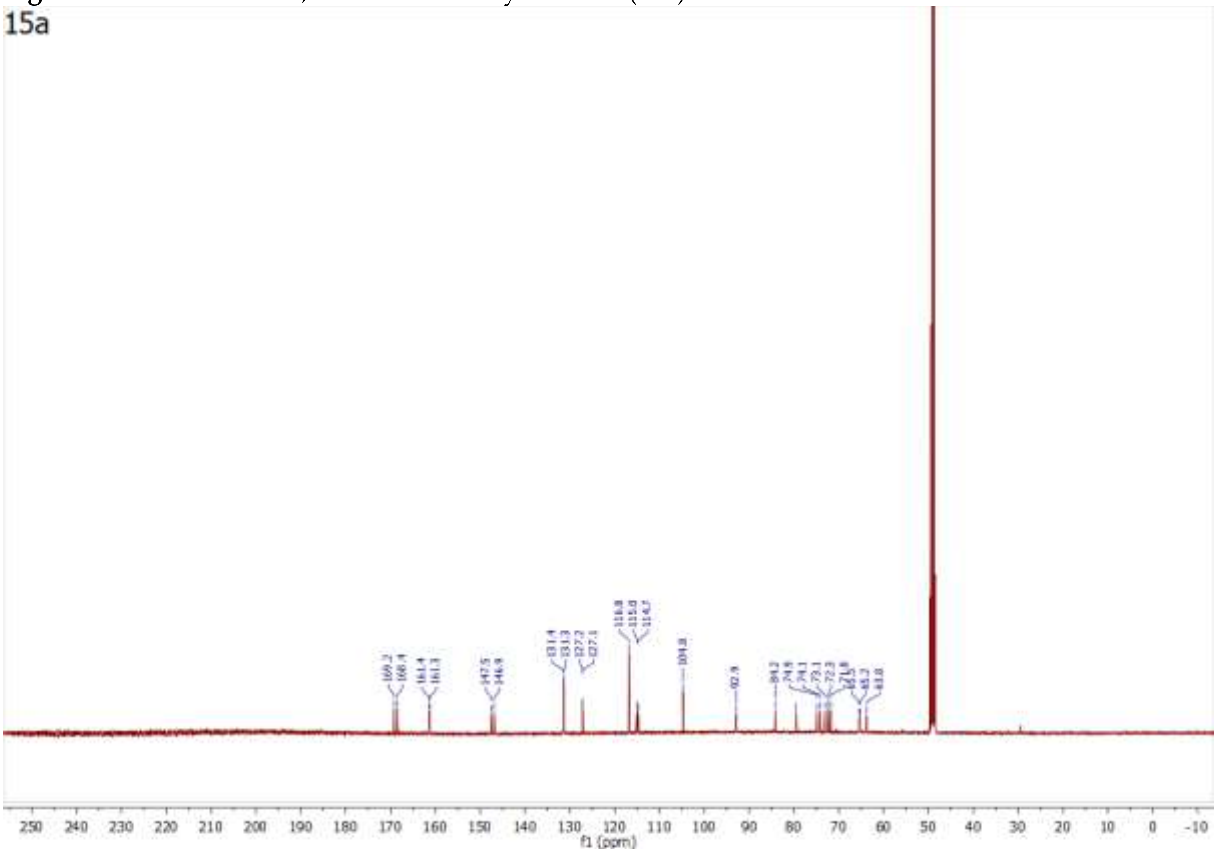

Figure S25. ^{1}H NMR of 6-*O*-feruloyl-3'-*O*-coumaroyl sucrose (**15b**)

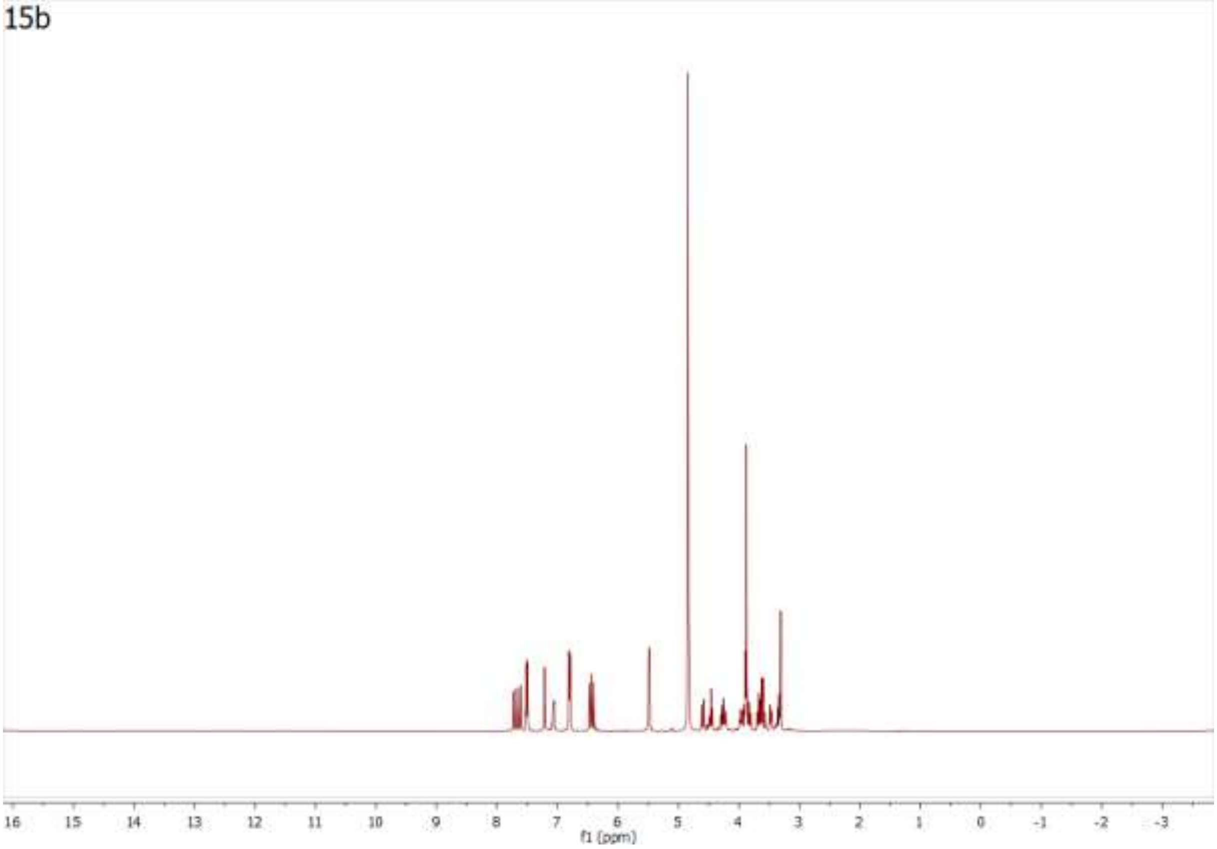

Figure S26. ^{13}C NMR of 6-*O*-feruloyl-3'-*O*-coumaroyl sucrose (**15b**)

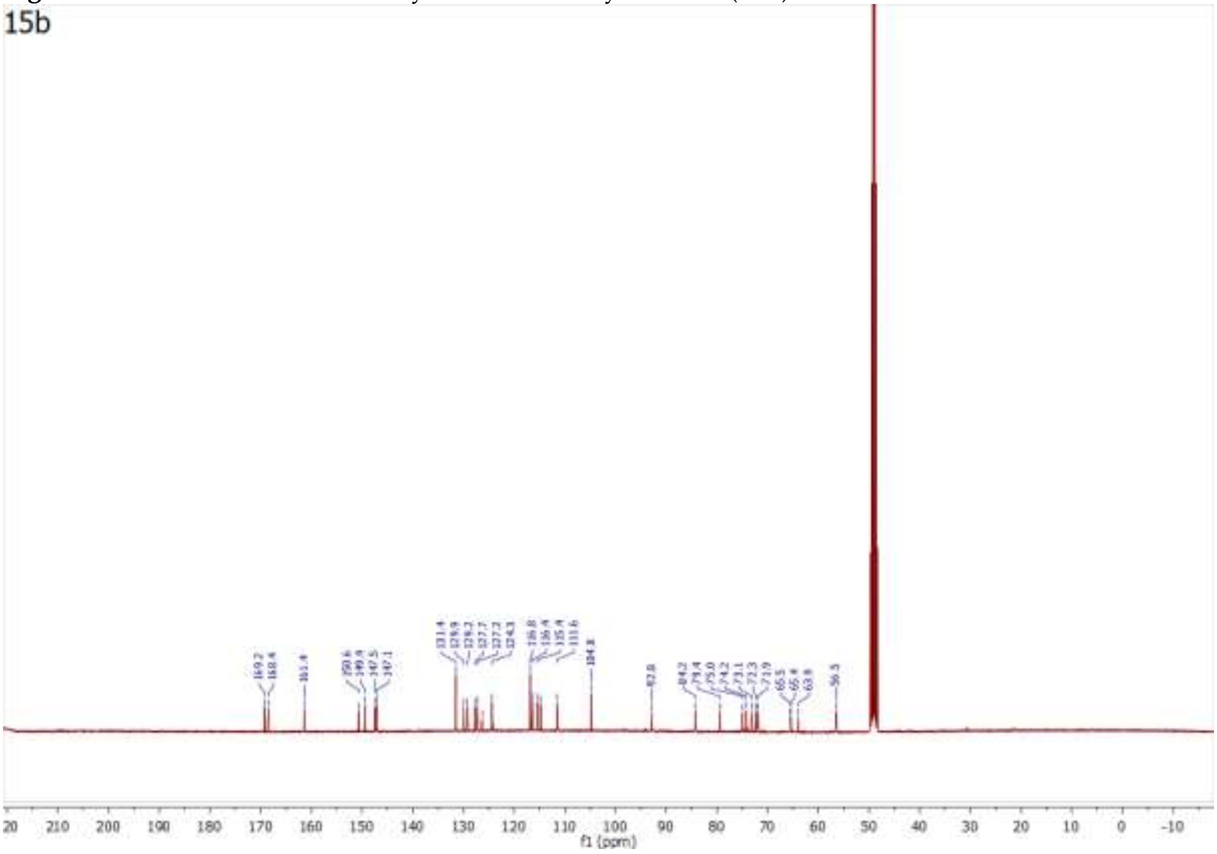

Figure S27. ^{1}H NMR of 6,3'-di-*O*-(3,4,5-tri-*O*-methoxycinnamoyl) sucrose (**15c**)

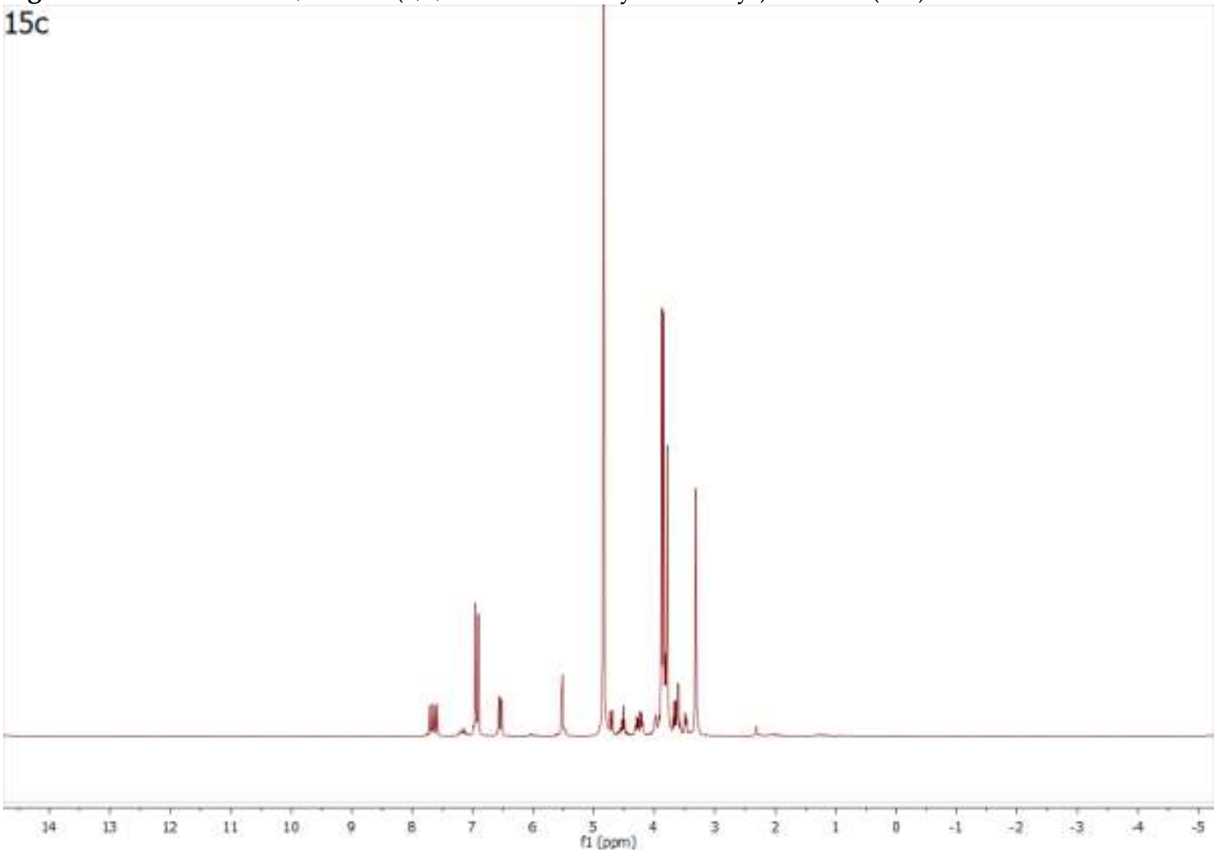

Figure S28. ^{13}C NMR of 6,3'-di-*O*-(3,4,5-tri-*O*-methoxycinnamoyl) sucrose (**15c**)

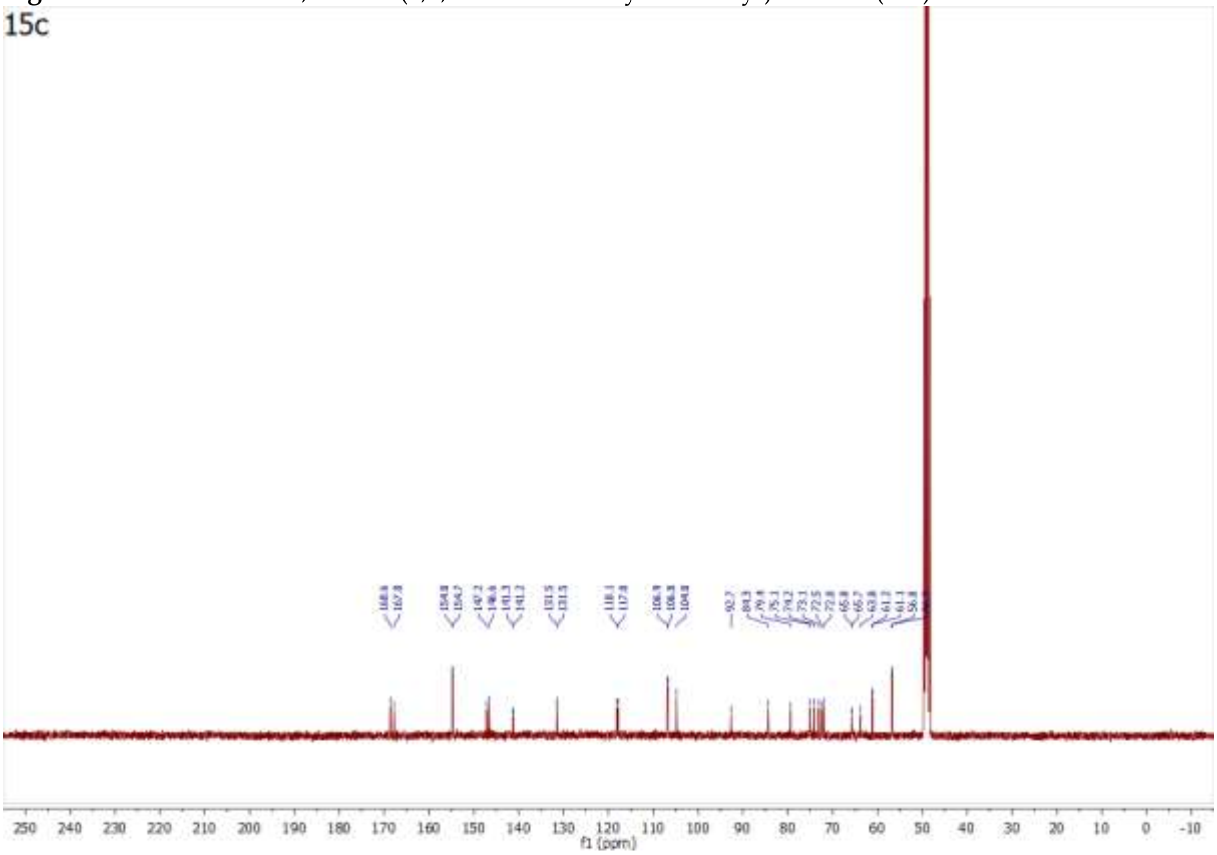

Figure S29. ^{1}H NMR of 6,3',4'-tri-*O*-coumaroyl sucrose (**17a**)

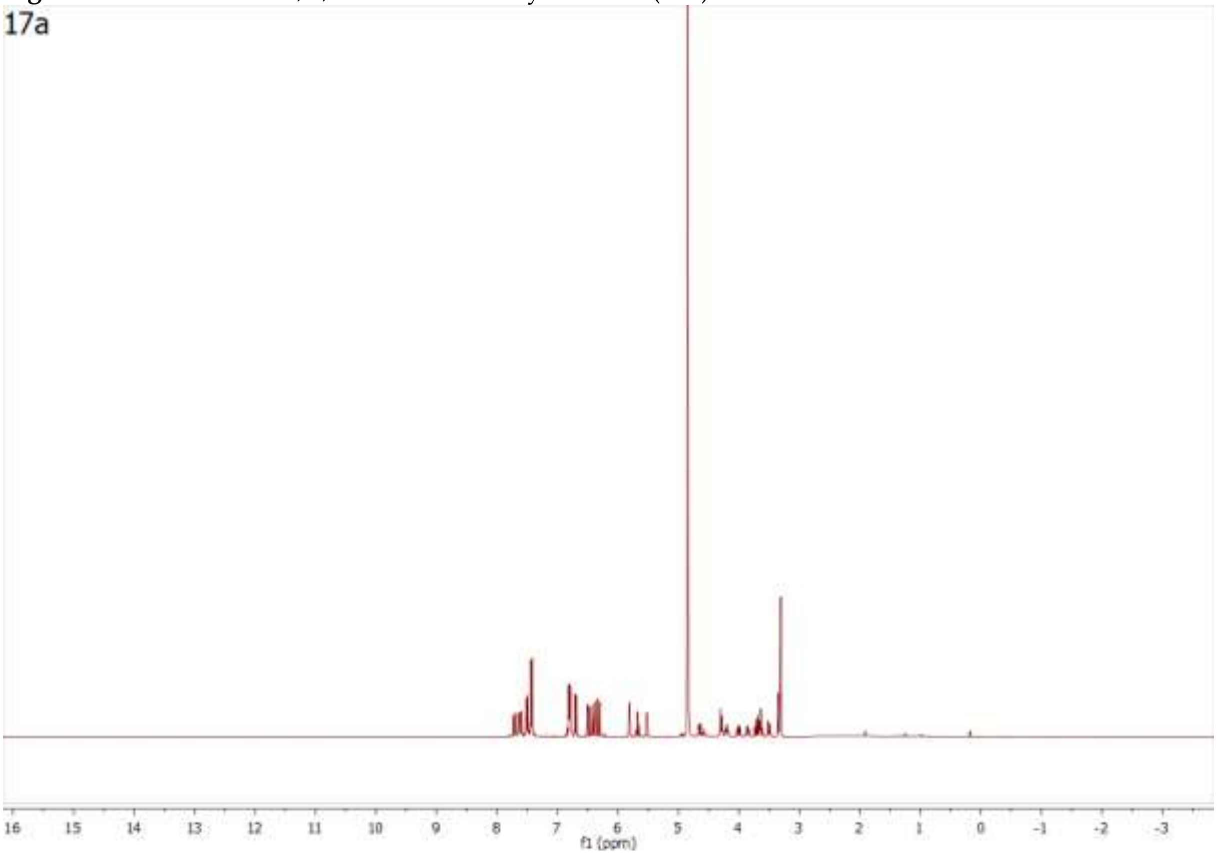

Figure S30. ^{13}C NMR of 6,3',4'-tri-*O*-coumaroyl sucrose (**17a**)

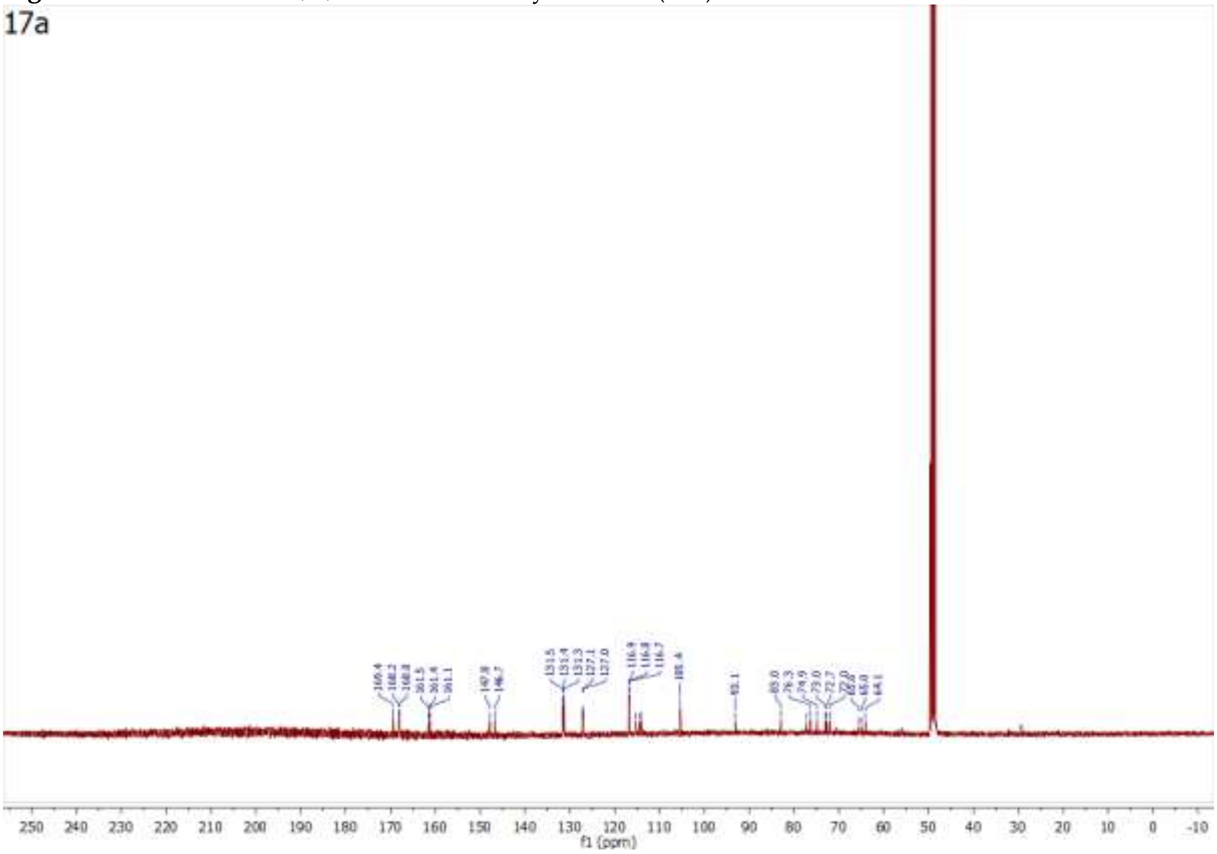

Figure S31. ^{1}H NMR of 6,3',6'-tri-*O*-coumaroyl sucrose (**18a**)

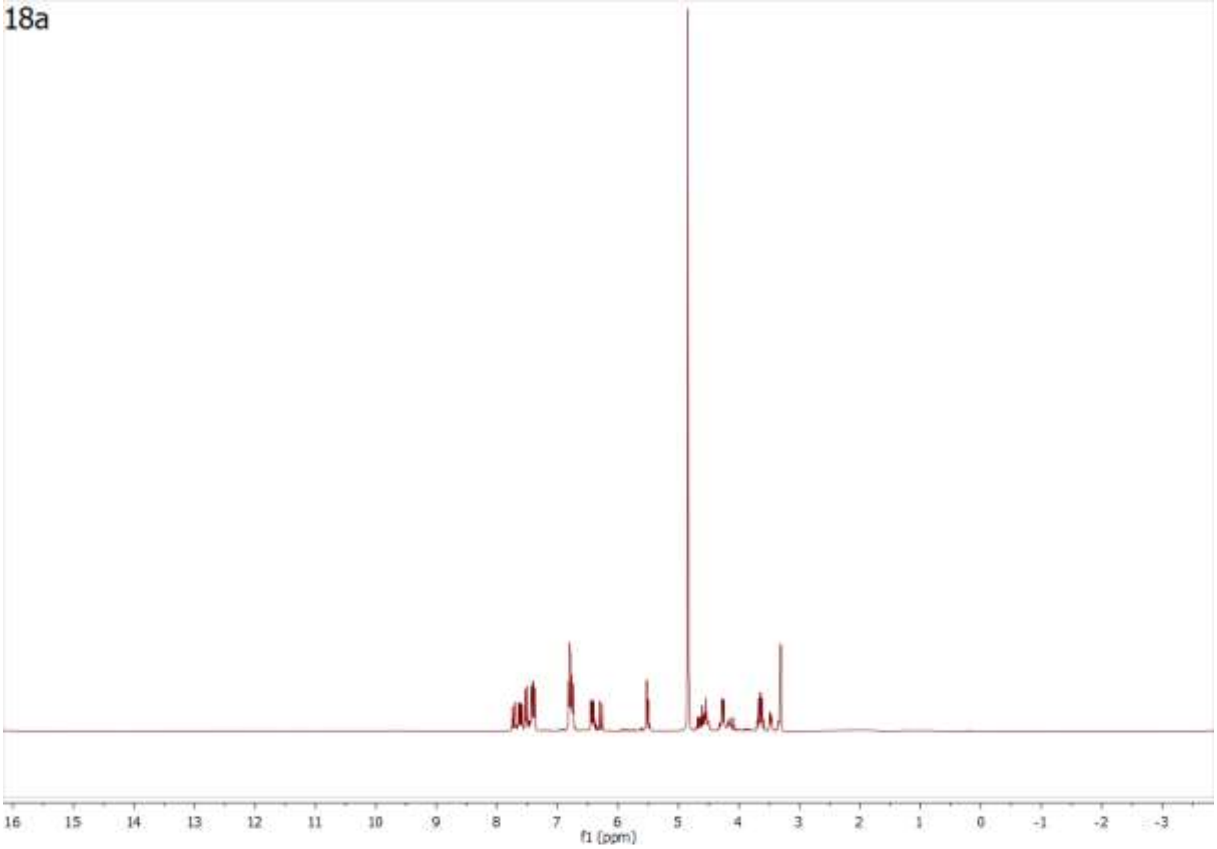

Figure S32. ^{13}C NMR of 6,3',6'-tri-*O*-coumaroyl sucrose (**18a**)

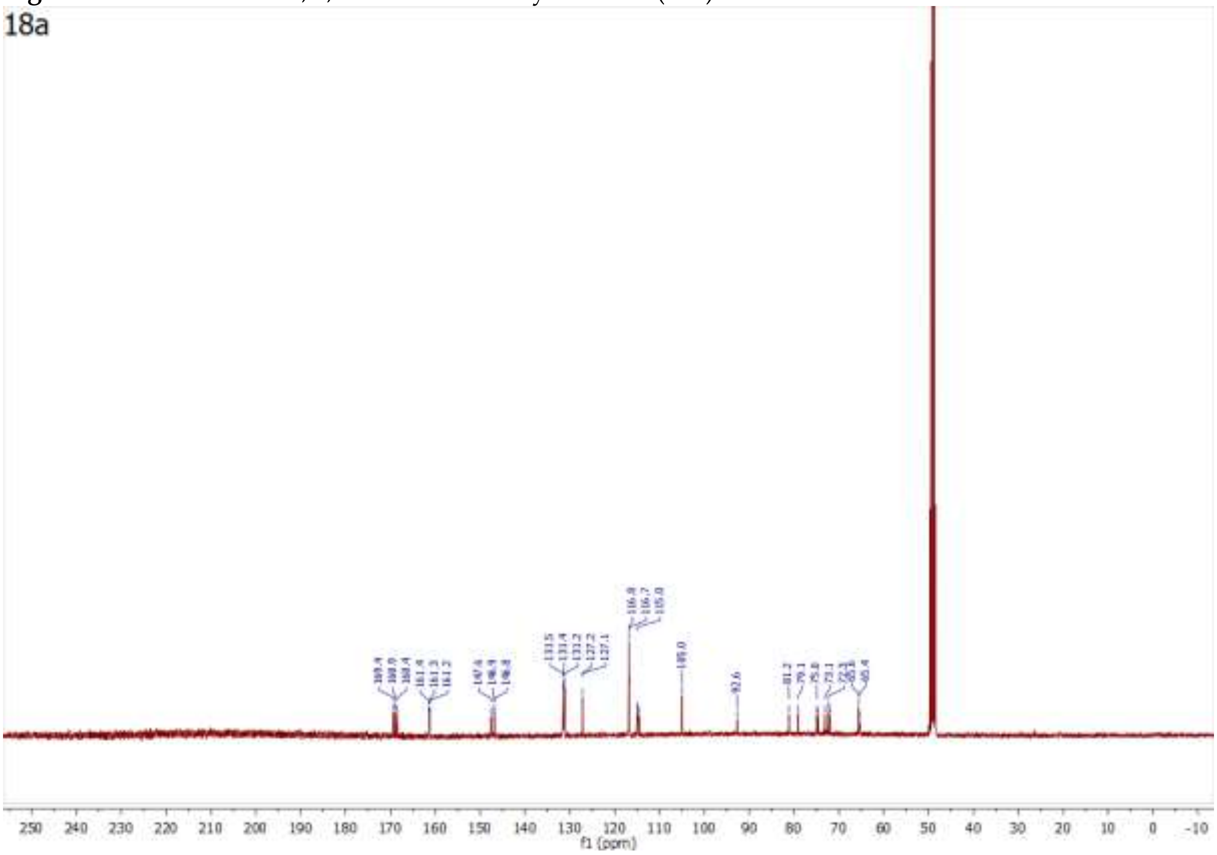

Figure S33. ¹H NMR of 6,6'-di-*O*-feruloyl-3'-*O*-coumaroyl sucrose (**18b**)

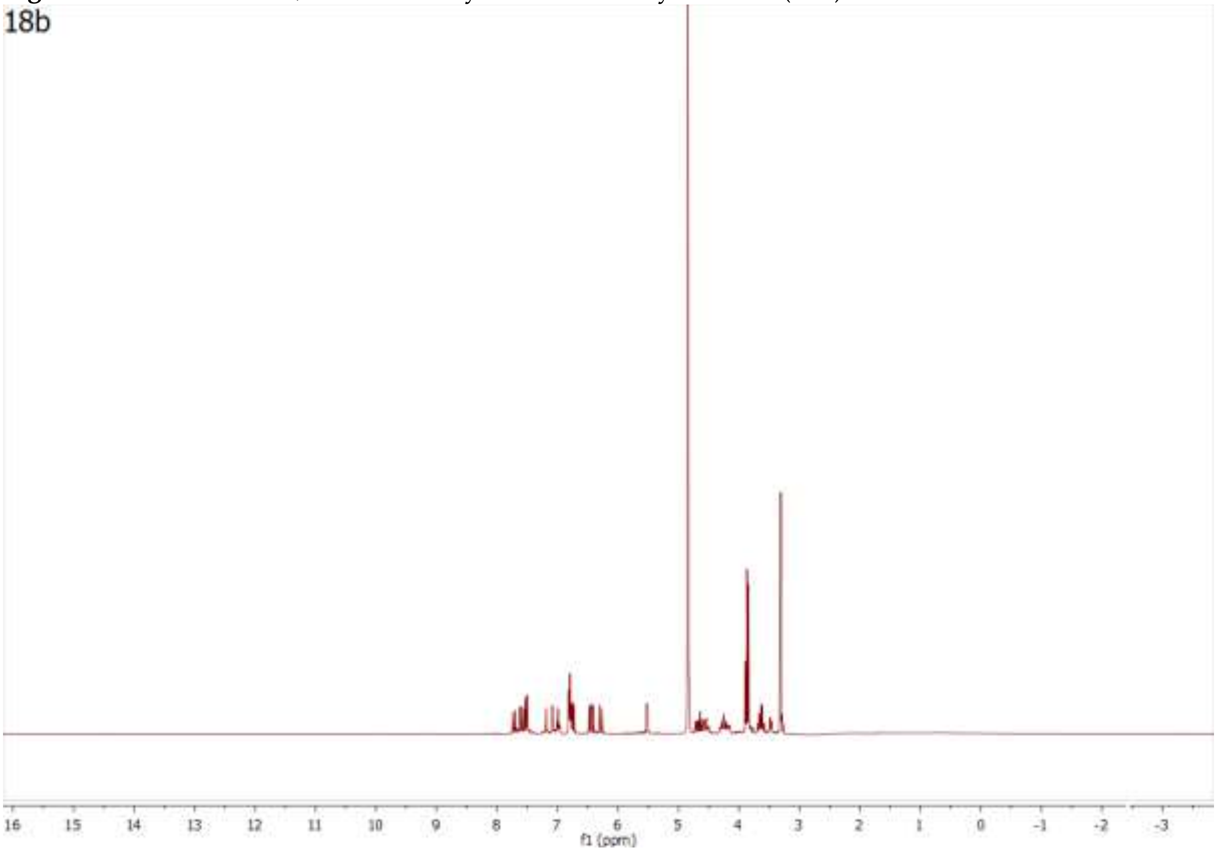

Figure S34. ¹³C NMR of 6,6'-di-*O*-feruloyl-3'-*O*-coumaroyl sucrose (**18b**)

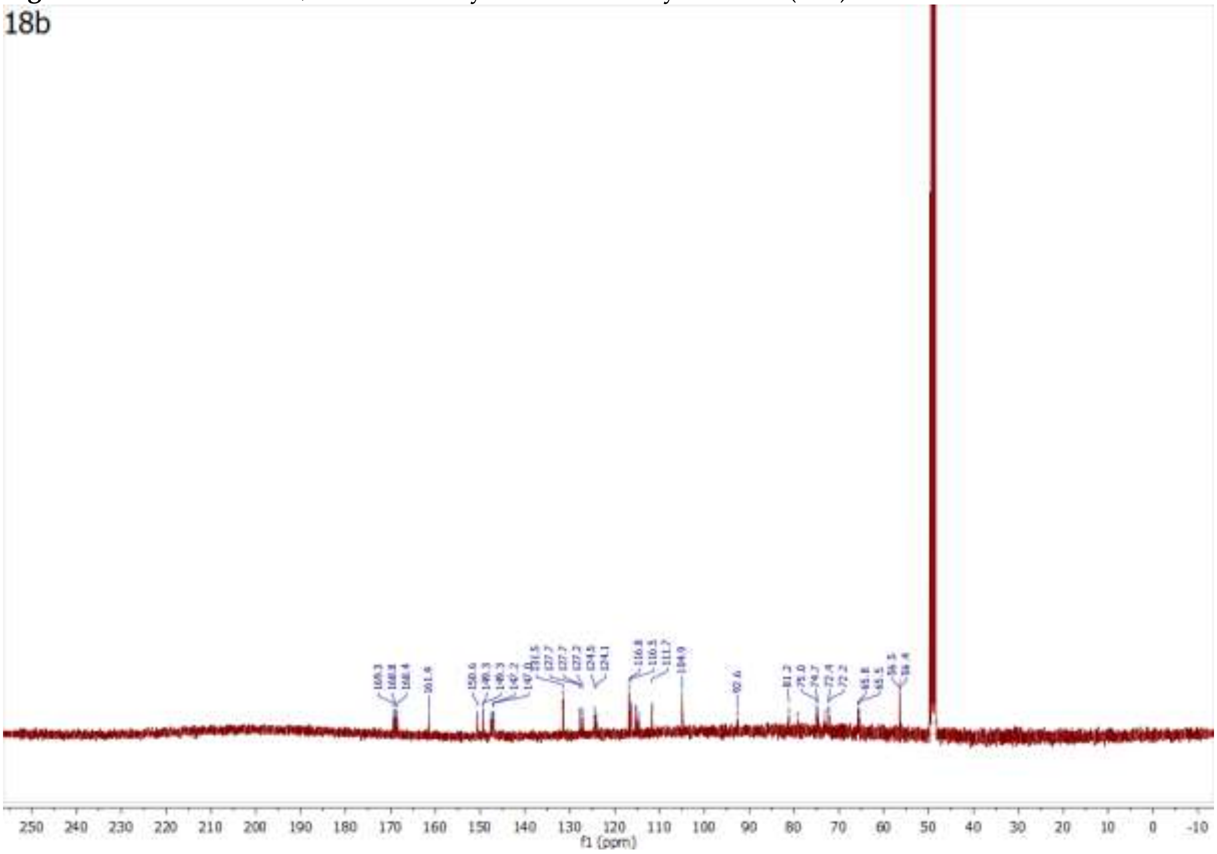

Figure S35. ¹H NMR of 2,6-di-*O*-(3,4,5-tri-*O*-methoxycinnamoyl) sucrose (**20**)

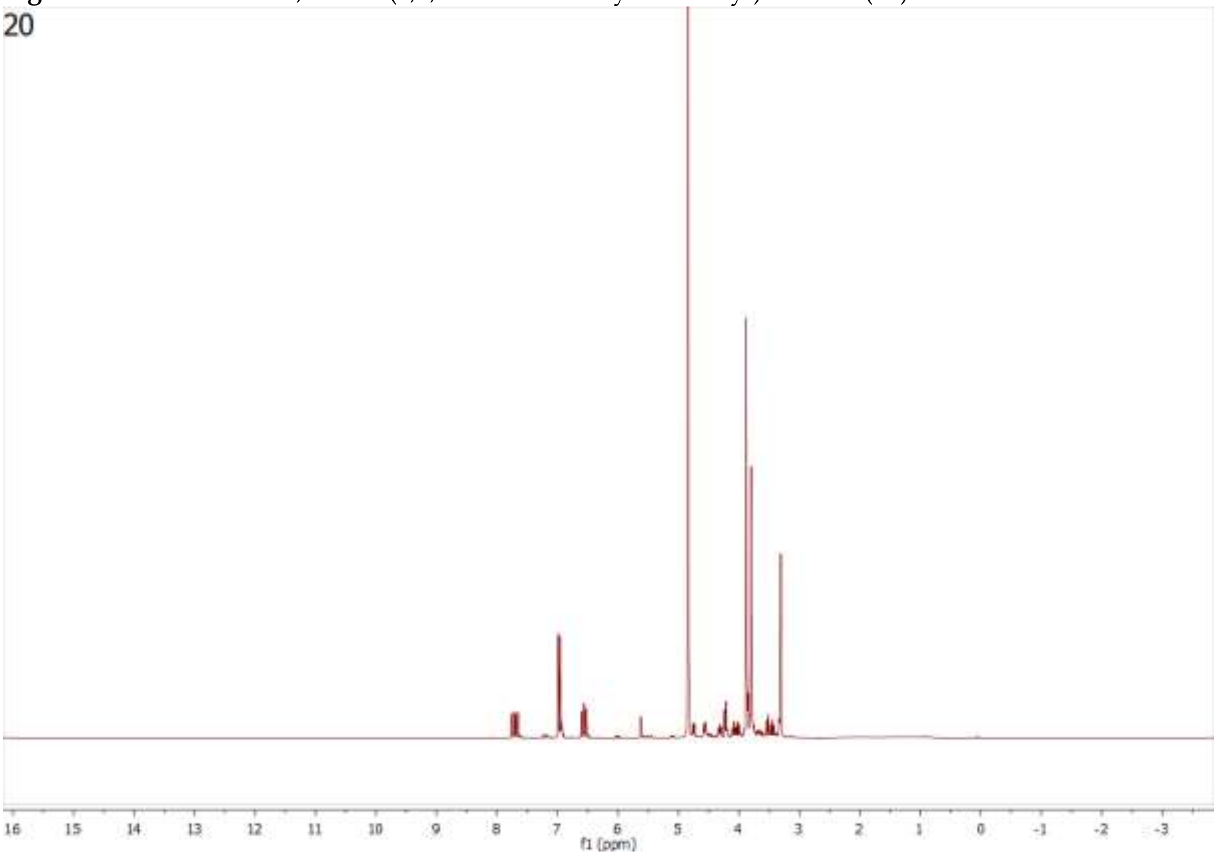

Figure S36. ¹³C NMR of of 2,6-di-*O*-(3,4,5-tri-*O*-methoxycinnamoyl) sucrose (**20**)

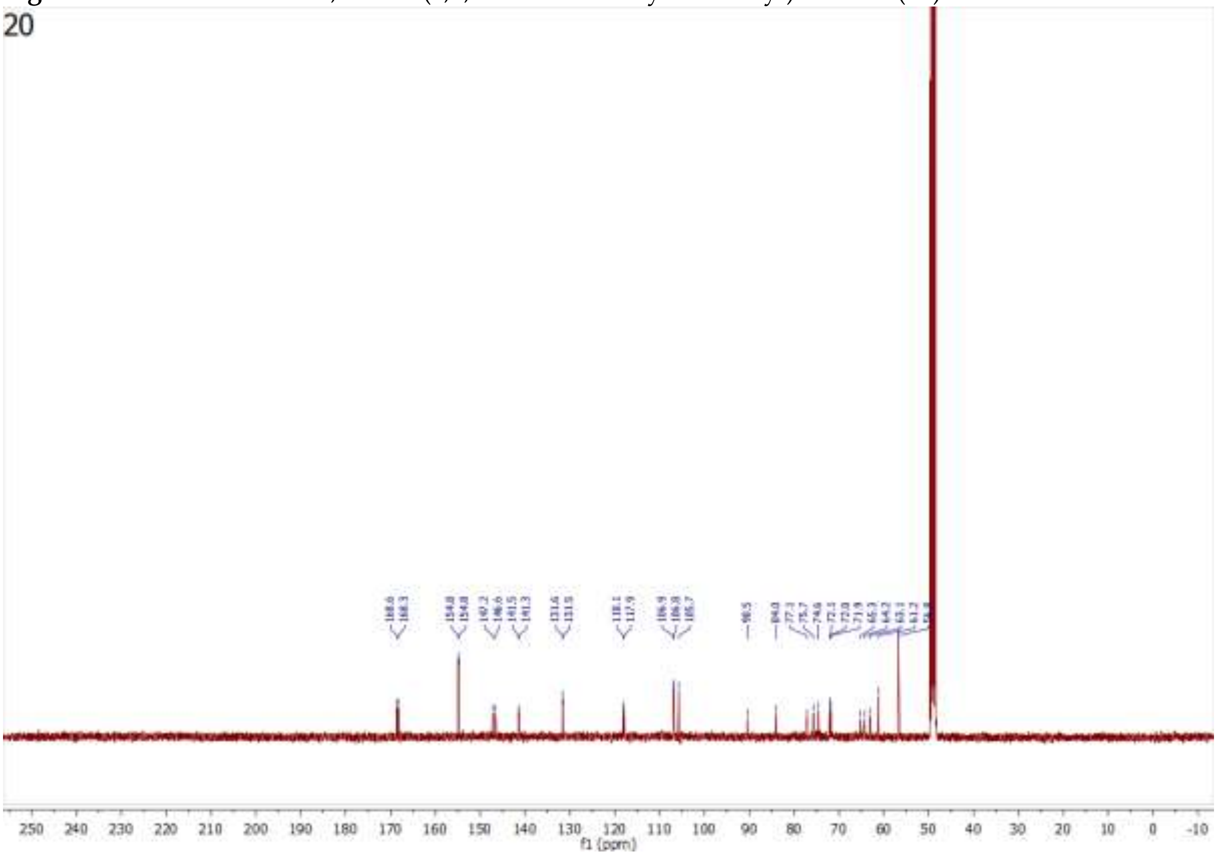

Supplement: Supplementary file 1 [file molecules-29-04067-s001.zip › molecules-3172798-supplementary.pdf]
